# Detecting photoinduced symmetry-breaking charge separation with near-zero driving force in a perylene diimide cage

**DOI:** 10.1039/d6cp02527g

**Published:** 2026-08-03

**Authors:** Estefanía Sucre-Rosales, Ricardo J. Fernández-Terán, Hsin-Hua Huang, Tomáš Šolomek, Eric Vauthey

**Affiliations:** a Department of Physical Chemistry, University of Geneva, 30 Quai Ernest-Ansermet CH-1211 Geneva 4 Switzerland eric.vauthey@unige.ch; b Department of Chemistry, University of Basel St. Johanns-Ring 19 CH-4056 Basel Switzerland; c Van’t Hoff Institute for Molecular Sciences (HIMS), University of Amsterdam P.O. Box 94157 1090 GD Amsterdam The Netherlands t.solomek@uva.nl

## Abstract

Photoinduced symmetry-breaking charge separation (SB-CS) between two identical molecules has been reported with an increasing number of chromophores, but is sometimes difficult to detect when reversible. Using a perylenediimide-based molecular cage, we demonstrate that the combination of electronic transient absorption and time-resolved fluorescence enables the detection of SB-CS even when no clear spectral signature of the ionic product is visible in the transient spectra. This is possible thanks to the presence of the easily observable delayed fluorescence and the global target analysis of both transient absorption and time-resolved fluorescence data using a modified exciplex model. We found that SB-CS in this cage occurs reversibly in a broad range of solvent polarities, even in highly polar media. On the basis of molecular dynamics simulations, the weak driving force of SB-CS is explained by the limited mobility of the solvent molecules inside the cage, and the reduced solvation energy of the charge-separated state that results.

## Abstract 2

Combined transient absorption and time-resolved fluorescence measurements of a PDI Cage allow for detecting reversible symmetry breaking charge separation in a broad range of solvent polarity.

## Graphical abstract

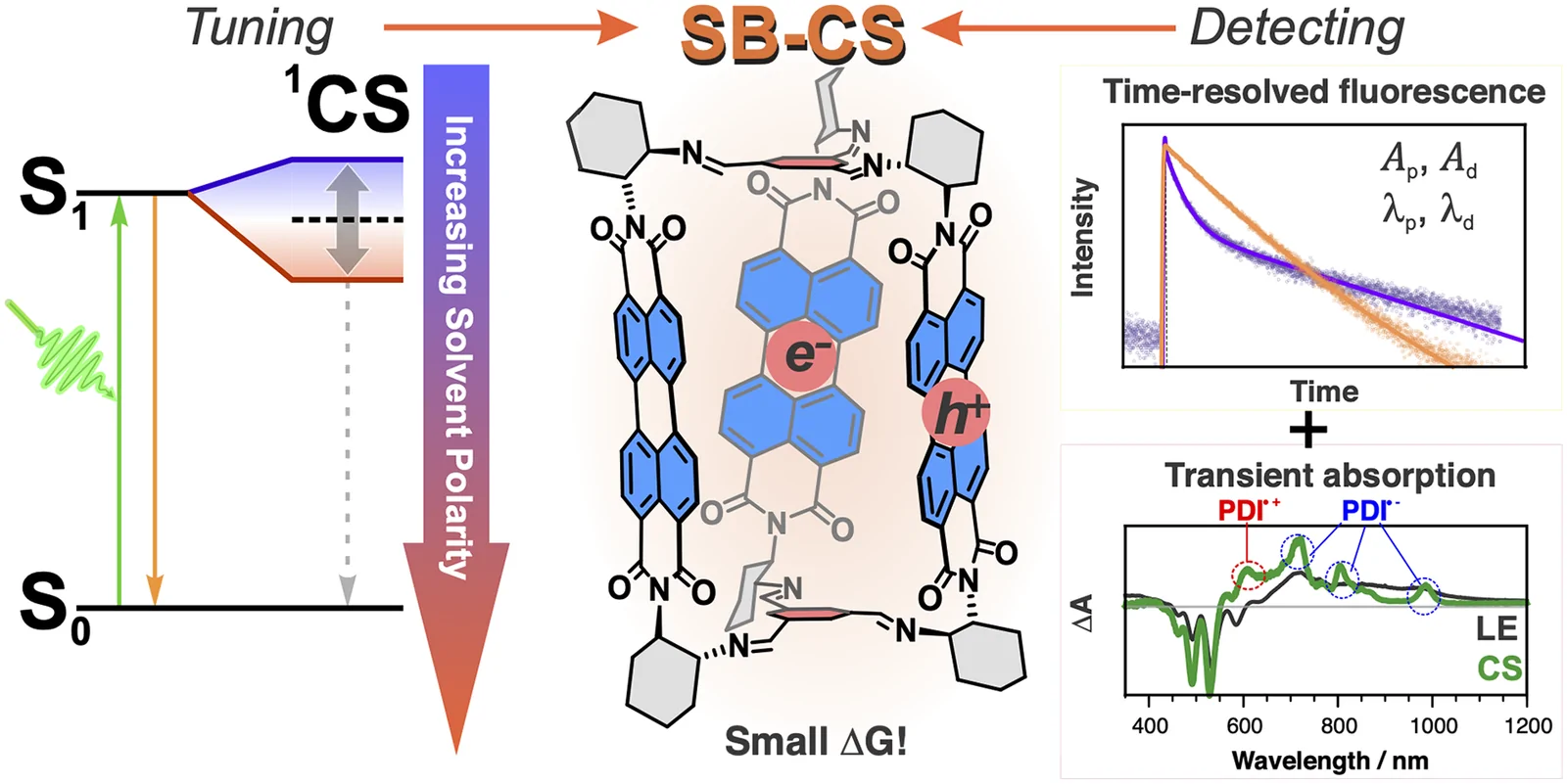

## 1. Introduction

Photoinduced symmetry-breaking charge separation (SB-CS) has recently attracted considerable attention as a promising alternative to achieve charge separation with low thermal loss and slow recombination; desired features for many applications including energy harvesting and conversion,^1,2^ as well as photocatalysis.^3,4^ This process, which has been reported with a increasing number of molecules,^1,2,5–20^ consists of a photoinduced electron transfer between two identical chromophores, one them in an electronic excited state, and, thus, leads to the population of a charge-separated (CS) state.^21,22^ A-priori, there should be no driving force for such SB-CS, *i.e.* Δ*G*_SBCS_ ≃ 0, because the optical gap can often be crudely approximated to the HOMO–LUMO gap, which is itself related to the sum of the reduction and oxidation potentials. However, when properly accounting for the electrostatic interactions in the description of the electronic excited state,^23^ and for the solvation energy of the CS state, it appears that, in polar media, Δ*G*_SBCS_ is either slightly positive or negative, depending on a subtle balance between various molecular parameters. Solvent stabilisation of the product plays indeed a decisive role in enabling SB-CS.^19,24,25^ In non-polar media, the charge-separated (CS) state is usually above the locally-excited (LE) state, *i.e.* Δ*G*_SBCS_ >0, and SB-CS is not operative.^21,26^

Perylene diimide (PDI) chromophores constitute a versatile platform for investigating SB-CS due to their strong absorption, high photostability, and appropriate redox properties.^27–32^ PDI-based multichromophoric architectures were used to investigate the influence of electronic coupling and solvation energy on the branching between SB-CS and other competitive pathways, such as excimer formation.^22^ Electronic coupling between the sub-units can be controlled by varying the length of the covalent bridge.^11,15,33–35^ It was found that in highly coupled systems, excimer formation may compete with or mediate charge separation,^34,36^ whereas a reduced electronic coupling upon increasing the interchromophoric distance can suppress excimer formation and enable SB-CS.^37^ The effect of solvation was investigated using shape-persistent systems, where interchromophoric distance and electronic coupling are fixed. Solvent polarity was found to allow for a fine tuning of the thermodynamics and the kinetics of SB-CS.^13,15,38^

**Fig. 1:**
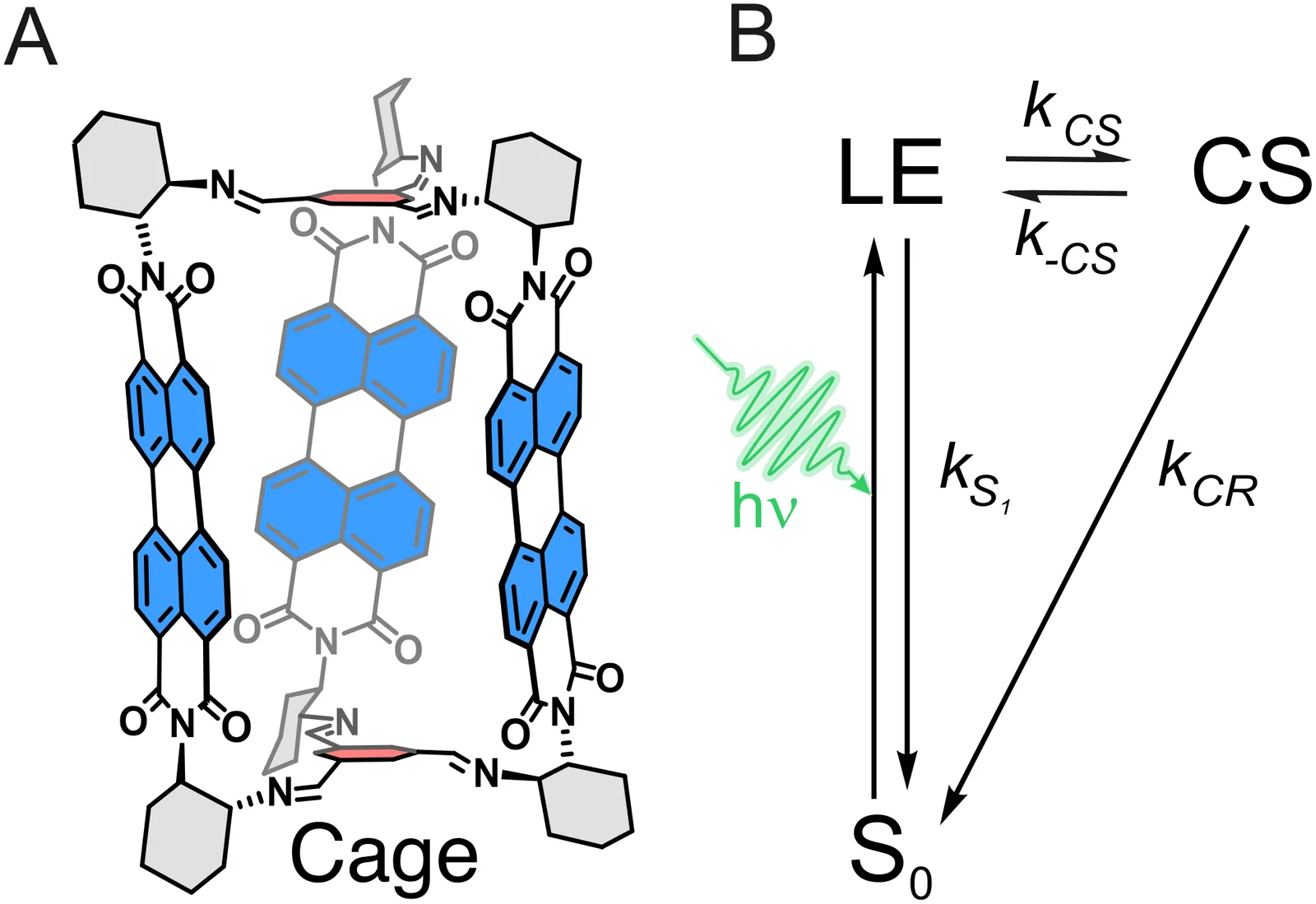
(A) Structure of Cage (left). (B) Minimal model for reversible symmetry-breaking charge separation (right).

We recently reported on a molecular cage (Cage, Fig. 1A) consisting of three covalently linked PDI units.^14^ Whereas in a weakly polar solvent like toluene (TOL),^39^ the fluorescence decay of Cage was found to be monoexponential, pointing to an electronic excitation localised on a single PDI unit, delayed fluorescence was detected in medium to highly polar solvents, such as dichloromethane and benzonitrile (BCN). This behaviour was attributed to the presence of an equilibrium between the emissive locally-excited (LE) S_1_ state and a non-emissive intermediate stabilised in polar environments. Transient absorption (TA) spectroscopy measurements in BCN allowed for the observation of spectral features consistent with the PDI radical cation, PDI˙^+^, while population of the triplet state was excluded. These observations suggested the population of a CS state *via* SB-CS. However, previous studies,^40^ have shown that similar spectral features could also originate from a correlated triplet-pair state resulting from singlet fission. Moreover, the limited spectral window of the UV-Vis TA measurements prevented unambiguous detection of the PDI radical anion, PDI˙^−^, whose absorption extends into the near-infrared (NIR).^14^ As a result, the definitive confirmation of SB-CS and the quantitative determination of the associated rate constants remained unresolved.

Beyond the spectroscopic identification of the CS state, a second challenge lies in the quantitative determination of each elementary kinetic step. As illustrated in Fig. 1B, the minimal mechanistic scheme involves four independent rate constants, accounting for the equilibrium between the LE and SB-CS states, and their decay to the ground state. Given this complexity, conventional global target analysis often leads to mathematically non-unique solutions, as different combinations of rate constants may reproduce the TA data equally well.^41^ Without independent physical constraints, extraction of reliable kinetic parameters becomes inherently ambiguous.^42,43^

In the present work, we address both the spectroscopic and kinetic limitations of our previous study. By extending TA measurements into the NIR region, we can directly detect unambiguous spectral signatures of PDI˙^−^ and thereby confirm occurrence of SB-CS in highly polar media. Furthermore, by employing toluene (TOL) and benzonitrile (BCN) mixtures, we systematically tune solvent polarity and modulate the SB-CS dynamics. We apply a constraint-based target analysis of the TA data that integrates independent observables derived from time-resolved fluorescence measurements. This reduces the effective parameter space and allows obtaining unique, physically meaningful sets of rate constants. With this approach, we can detect occurrence of SB-CS in weakly polar environments, where the equilibrium is strongly on the LE state side and therefore the population of the CS state is hardly visible in the TA data. This approach is not limited to SB-CS, but can be extended to any case involving an equilibrium between an emissive and a longer-lived dark state, such as TADF systems.

## 2. Results and discussion

### 2.1. Transient absorption and time-resolved fluorescence

**Fig. 2:**
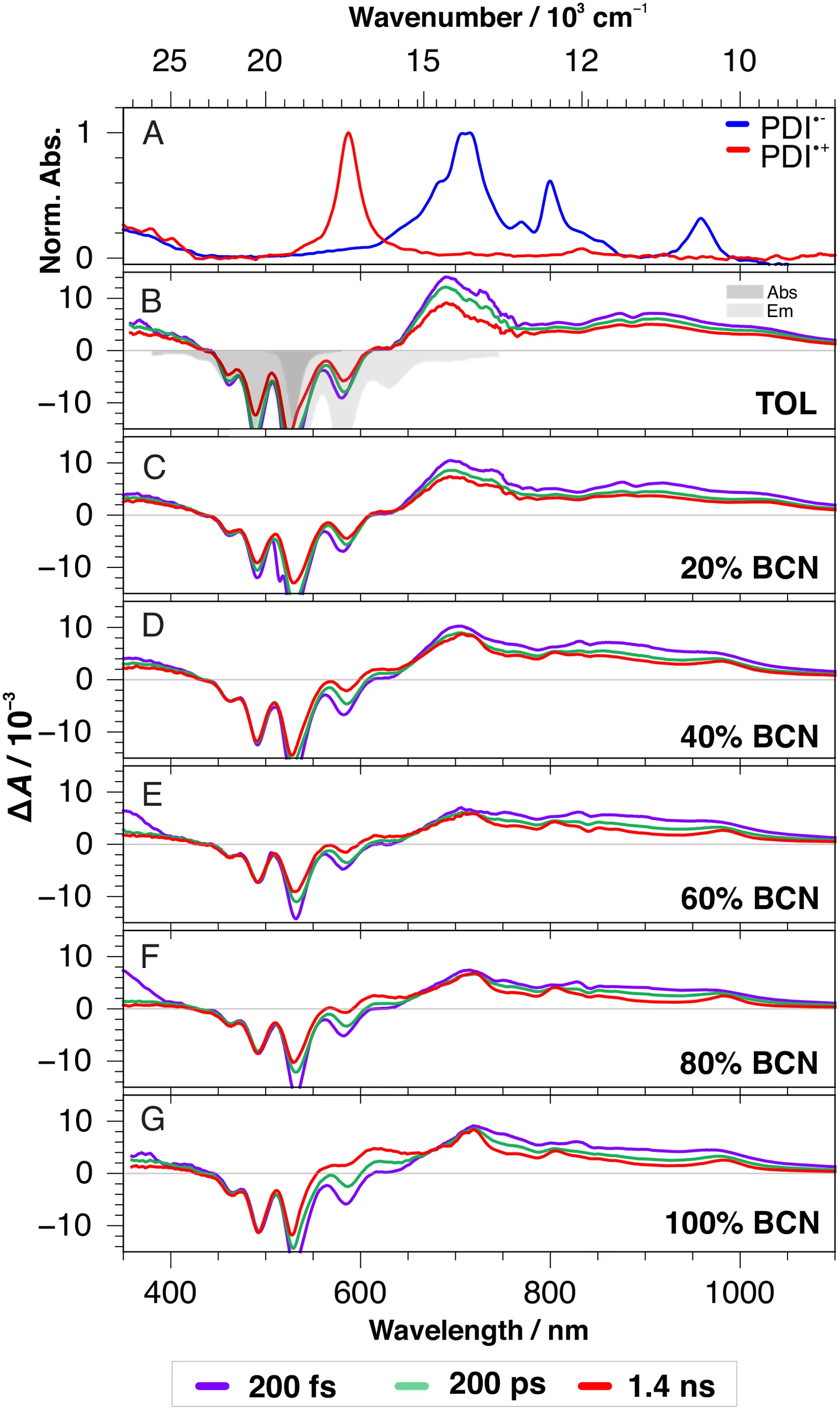
(A) Absorption spectra of PDI˙^+^ and PDI˙^−^ from spectroelectrochemistry experiments. (B–G) Transient absorption spectra of Cage in toluene (TOL)/benzonitrile (BCN) solvent mixtures of increasing polarity.

The TA dynamics measured with Cage in TOL are very similar to those recorded with the PDI monomer (Fig. 2 and Fig. S7 and S8),^44^ with the negative and structured band originating from both ground-state bleach (GSB) and stimulated emission (SE), the broad excited-state absorption (ESA) band extending from 650 to 1150 nm, and the onset of another ESA band below 400 nm. The TA data can be reproduced using the sum of two exponential functions, with the faster component assigned to the relaxation of the Franck–Condon (FC) state to the equilibrium S_1_ state, and the slower ns component attributed to the decay of the S_1_ state population (Fig. S12). This is in agreement with the TCSPC measurements, which can be reproduced using a single exponential decay with a 5.5 ns lifetime (Fig. S6 and Table S2). Given the strong similarity of the S_1_–S_0_ absorption and emission bands of Cage and PDI monomer,^14^ this excitation should be mostly localised on a single PDI unit of Cage and will henceforth be designated as LE state.

The early TA spectra measured with Cage in BCN resemble those in TOL, pointing to the population of the LE state (Fig. 2G). However, this spectrum transforms on a few hundreds of ps to show the GSB band and four positive bands, around 615, 720, 810 and 985 nm. As illustrated in Fig. 2A and Fig. S5, the 615 nm band can be attributed to PDI˙^+^,^45^ whereas the others can be assigned to PDI˙^−^.^14^ The simultaneous presence of the spectral signatures of both radical ions provides direct spectroscopic signature SB-CS in this solvent.

The polarity of the environment was then varied incrementally between those of TOL and BCN using mixtures of these two solvents. The ultrafast Vis-NIR TA data in 20% BCN are very similar to those in pure TOL (Fig. 2B and C). However, the visible TA spectra measured on a longer timescale reveal that the SE band, *i.e.*, the population of the LE state decays faster than in pure TOL. Clearer spectroscopic signatures of the CS state become only discernible at ≥40% BCN.

**Fig. 3:**
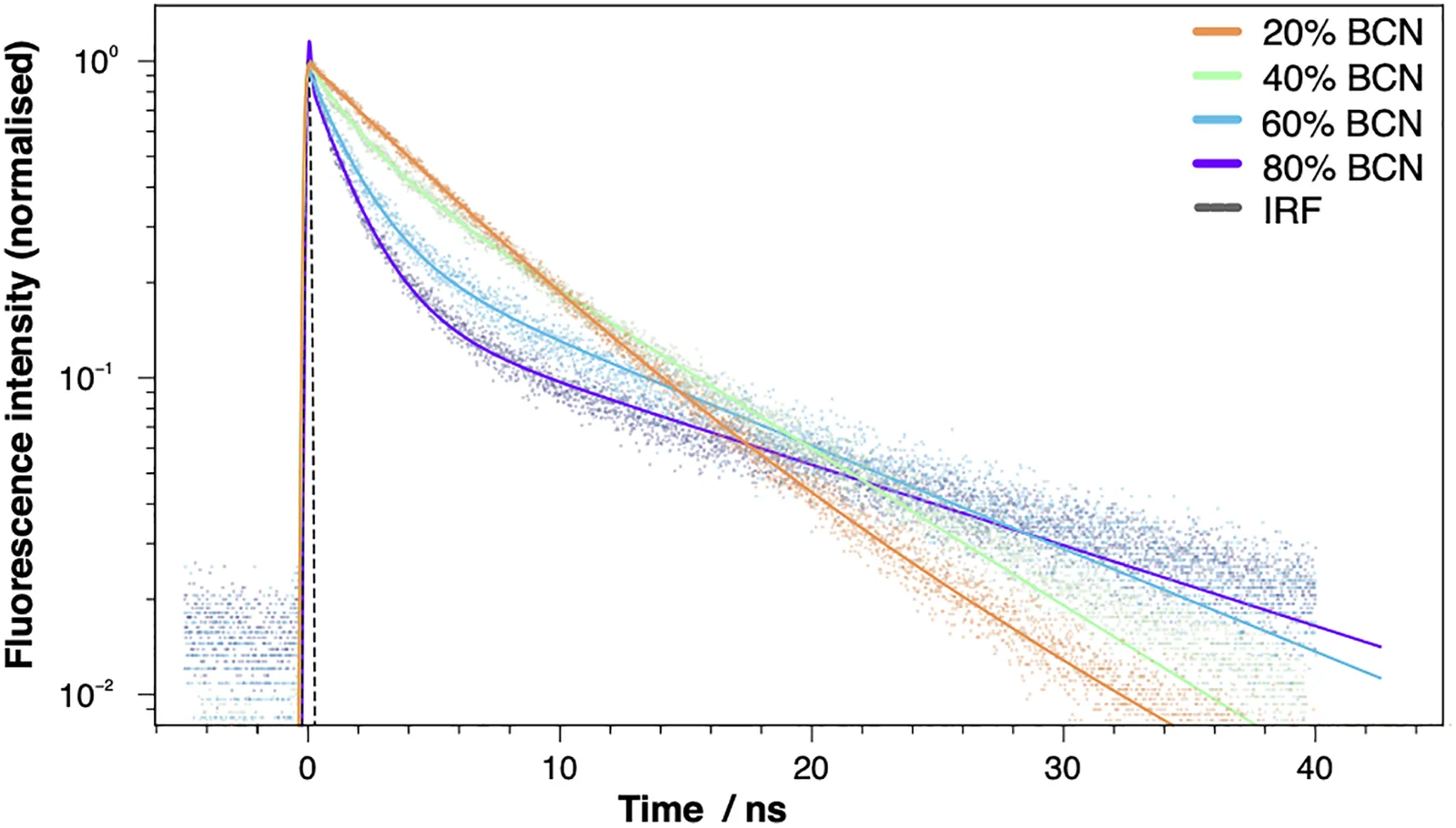
Time-correlated single-photon counting profiles recorded with Cage in toluene/benzonitrile mixtures and best-fits of the convolution of a biexponential function with the instrument response function (IRF).

The fluorescence decay at ≥20% BCN requires the sum of two exponential functions to be properly reproduced (Fig. 3). The time constant of the slow component increases with the volume of BCN, whereas its relative amplitude decreases (Table S2).

In principle, the TA data in pure BCN and in TOL/BCN mixtures can be well reproduced assuming a series of successive exponential steps. However, such simple analysis does not properly capture the photodynamics of Cage, because it does not account for the equilibrium between the LE and the CS states, revealed by the presence of delayed fluorescence. The absence of delayed fluorescence in TOL is consistent with a CS state located energetically well above the LE state and not accessible at room temperature. A 20% BCN content stabilises the CS state sufficiently to allow for reversible SB-CS. The CS state is further stabilised as the BCN content of the mixture is increased. Consequently, the equilibrium becomes increasingly displaced toward the CS state as recombination to the LE state is slowed down, accounting for the weaker and slower delayed fluorescence.

### 2.2. Data analysis

These results illustrate the strength of combining TA and time-resolved fluorescence spectroscopies, the former enabling unambiguous identification of the SB-CS state and the latter allowing for a clear detection of delayed fluorescence and the presence of an equilibrium. Another advantage is that the TCSPC data can be used as constraints in the global analysis of the TA data.

**Fig. 4:**
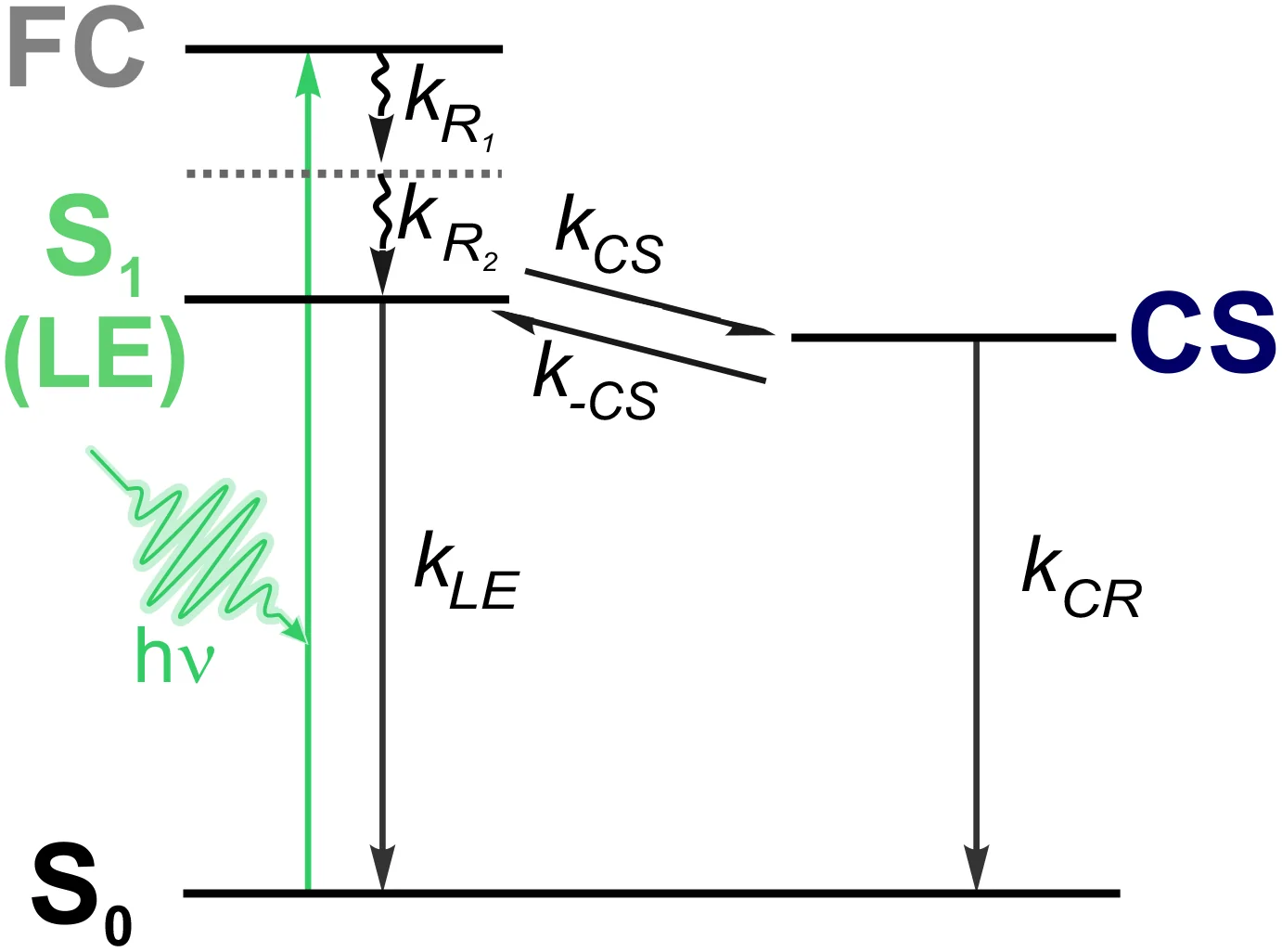
Target model used for the global constrained analysis of the transient absorption data recorded with Cage.

The scheme in Fig. 4 was used as a target model in the global analysis of the TA data. It bears a strong resemblance to the exciplex model of Ware,^46^ and therefore, the fluorescence decay, reflecting the time-evolution of the equilibrated LE population can be described in a similar fashion (see details in Section S2):

1 *I*_fl_(*t*) = *A*_p_ exp(*λ*_p_*t*) + *A*_d_ exp(*λ*_d_*t*)

with *λ*_i_ = −*k*_i_, *k*_p_ and *k*_d_ being the decay rate constants of prompt and delayed emission:2a
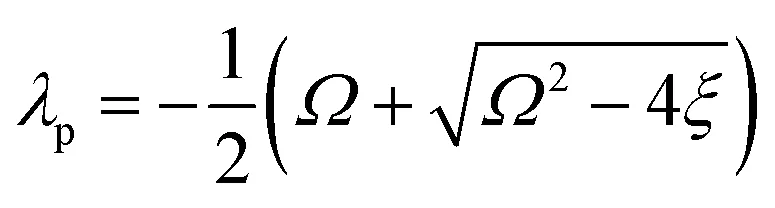
2b
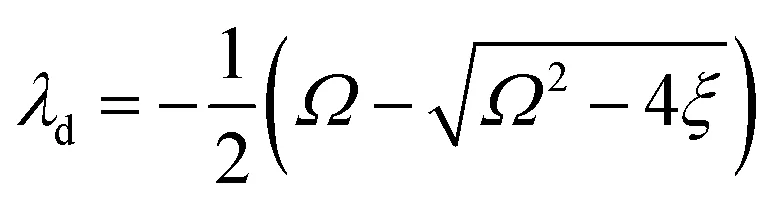
where *Ω* ≡ *k*_S1_ + *k*_CS_ + *k*_−CS_ + *k*_CR_, and *ξ* ≡ *k*_S1_(*k*_−CS_ + *k*_CR_) + *k*_CS_*k*_−CR_, and with *A*_p_ and *A*_d_ the amplitudes:3a
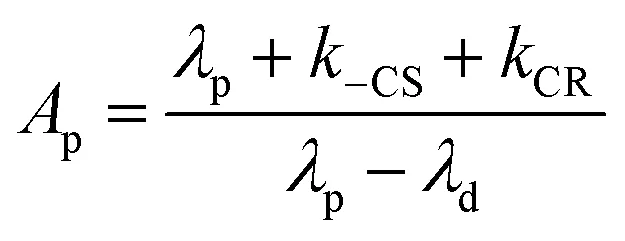
3b
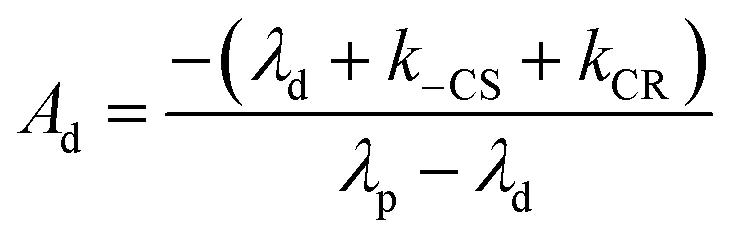


Therefore, the global analysis of the TA data using the scheme in Fig. 4 as target model was performed with the constraint that both eqn (2) and (3) are also fulfilled (see Section S2.3 for details). To limit the number of free parameters, the rate constant for the decay of the LE state to the ground state, *k*_LE_, was assumed to be close to that in TOL, where no SB-CS takes place, and was thus kept within (5–8 ns)^−1^.

Such exciplex-like model was already considered in the context of SB-CS. Holman *et al.* analysed TA time-profiles at a few wavelengths using eqn (1).^33^ As no constraint was imposed, unambiguous determination of the individual rate constants from the so-obtained *A*_i_ and *λ*_i_ values was not really possible. Wasielewski and coworkers improved this approach by first determining the *λ*_i_ values from a global biexponential analysis the TA data and, second, using the fluorescence quantum yield as constraint to determine the individual rate constants.^13,47^ However, this procedure yields evolution-associated difference absorption spectra, that are linear combinations of the TA spectra of the individual state/species, so-called species-associated difference absorption spectra (SADS).^41^ Our constrained global target analysis of the TA data offers the important advantage of yielding the SADS, which are very useful for testing the reliability of the analysis. To the best of our knowledge, such an approach was only used once before, but in a different context.^48^

**Fig. 5:**
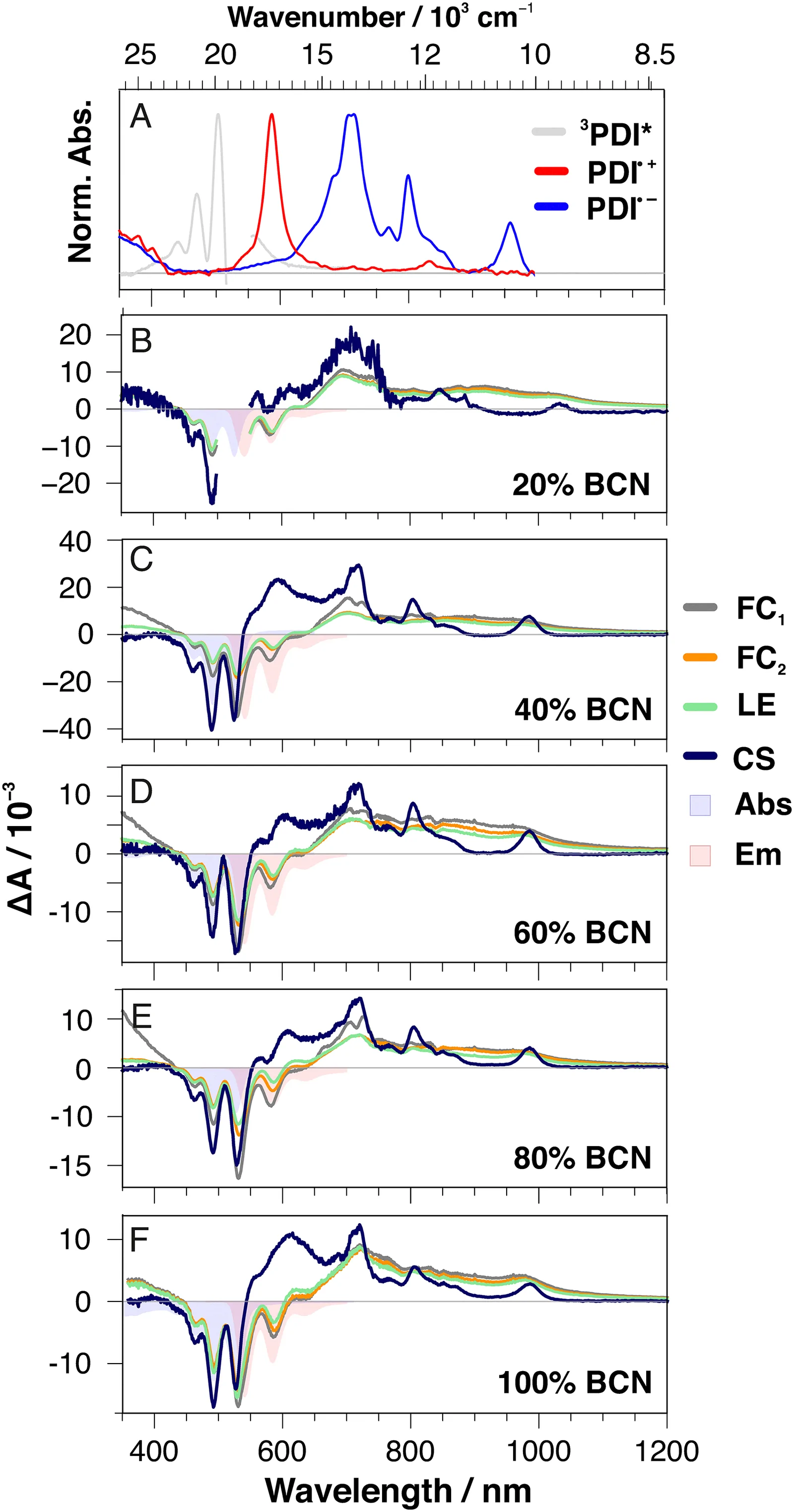
(A) Absorption spectra of ^3^PDI*,^51^ PDI˙^+^ and PDI˙^−^. (B–F) Species-associated difference absorption spectra obtained from a global analysis of the TA recorded upon excitation of Cage in TOL/BCN solvent mixtures of varying composition using the target model shown in Fig. 4 with the constraints imposed by eqn (2) and (3).

The model was first tested with BCN solutions, where occurrence of SB-CS is the most evident. The extracted time constants, *τ*_i_ = 1/*k*_i_, and the SADS are shown in Table 1 and Fig. 5, respectively.

**Table 1:** Relative amplitude of the prompt fluorescence, *A*_p_, time constants obtained from a constrained global analysis of the TA data measured with Cage in toluene/benzonitrile mixtures of different compositions,^a^ and driving force for SB-CS, Δ*G*_SBCS_, determined from the equilibrium constant between the LE and CS states, *K*_SBCS_ = *τ*_−CS_/*τ*_CS_

%BCN	*A*_p_	*τ*_CS_/ns	*τ*_−CS_/ns	*τ*_CR_/ns	Δ*G*_SBCS_/meV
20	0.30	17.6	3.2	65.0	44
40	0.35	9.0	2.6	44.3	30
60	0.66	4.1	4.4	30.0	2
80	0.78	3.2	5.3	44.5	−13
100	0.90	2.8	12.7	85.2	−40

a see Table S2 for a list of all parameters.

The first three SADS (FC_1_, FC_2_ and LE) are very similar, with the characteristic GSB, SE, and a broadband ESA that extends from 650 nm to 1200 nm. The ESA features in the first two SADS are slightly broadened compared to the third, as expected for a non-equilibrated excited state.^49^ The associated time constants, *τ*_R1_ and *τ*_R2_, should not be interpreted literally but reflect the non-exponential character of vibrational/structural/solvent relaxation dynamics, which occur on timescales ranging from a few hundreds of fs to a few tens of ps.^50^ The SADS attributed to the CS state shows the contributions of the GSB but not of the SE, as expected, and the distinct features of both PDI˙^+^,^45^ and PDI˙^−^,^14^ with the 615 and 720 nm bands having similar intensity. This is consistent with quantum-chemical calculations (see Section S3.4) predicting comparable extinction coefficients for the most intense band of both radicals. This analysis was repeated with the data recorded in TOL/BCN mixtures, and resulted in very similar SADS down to 40% BCN content (Fig. 5).

Since no spectral features of the triplet-state of PDI (Fig. 5A and S16) were observed in any of the SADS (Fig. 5), occurrence of processes, such as singlet fission or triplet charge recombination *via* spin–orbit charge-transfer ISC, can be safely excluded here.

The results obtained in the 20% BCN mixture deserves special attention. As noted before, whereas the TA spectra depicted in Fig. 2 exhibit almost no difference with those in pure TOL, the biexponential fluorescence decay suggests an equilibrium with a reversible SB-CS as in the more polar solvent mixtures (Fig. 3). Analysis using a simple sequential model clearly misses the population of the CS state even with a scheme where the decay of the CS state is faster than its population (Fig. S12C and D). By contrast, the spectral signatures of the CS state can be clearly identified when using the constrained target model, though some distortion is observed in the 650–720 nm region, where ESA and PDI˙^−^ bands overlap strongly. This result underlines the advantage of using such an approach to detect SB-CS in systems where clear identification of spectral signatures of the CS state is difficult.

### 2.3. Performance and limits of the model

**Fig. 6:**
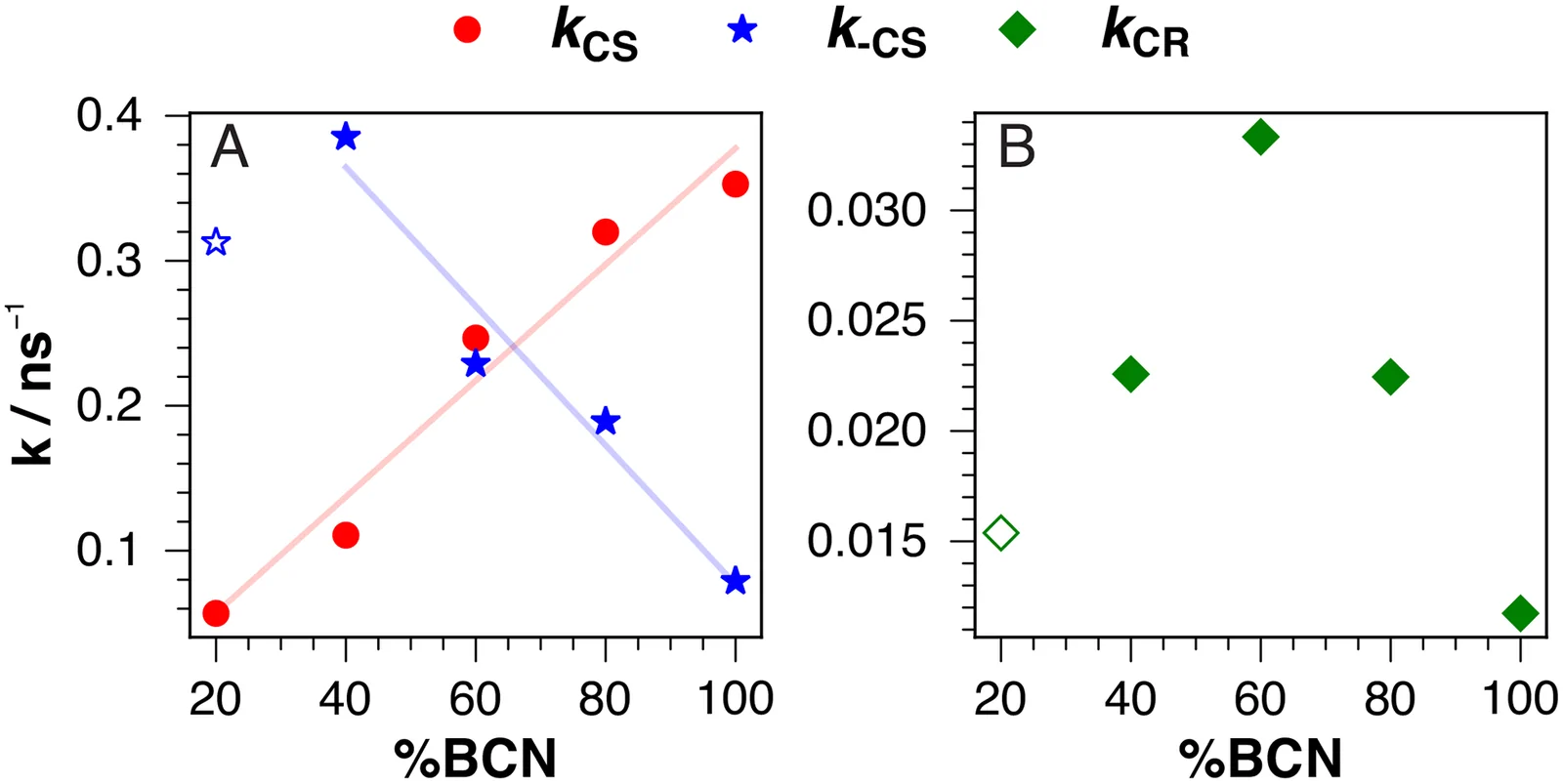
Rate constants of charge separation (*k*_CS_, A), of the reverse process (*k*_−CS_, A), and of charge recombination (*k*_CR_, B) as a function of the composition of the solvent mixture in % BCN (v/v). The empty markers correspond to values with a higher uncertainty. The lines serve as guides for the eyes.

The solvent dependence of the individual rate constants obtained from the above-described analysis is illustrated in Fig. 6. It appears that SB-CS accelerates continuously upon increasing the BCN content of the mixture with a rate constant *k*_CS_ increasing from (18 ns)^−1^ with 20% BCN to (2 ns)^−1^ in pure BCN. By contrast, *k*_−CS_ exhibits a continuous decrease, except at 20% BCN where a relatively small value is obtained from the analysis. Acceleration of SB-CS and slowing down of the reverse process are fully consistent with an increasing stabilisation of the SB-CS state relative to the LE state upon increasing solvent polarity, as discussed above. Although the constrained target analysis in 20% BCN is able to detect occurrence of SB-CS, the resulting rate constants should be considered with caution, given that the SADS of the CS state still contains contribution from the LE state. This suggests that, in these low polarity environments, the excited-state dynamics of Cage may not be fully accounted for by the scheme depicted in Fig. 4 (*vide infra*).

Finally, the rate constant for the charge recombination, *k*_CR_, does not exhibit a clear trend with the solvent polarity (Fig. 6B), but is always an order of magnitude smaller than *k*_CS_ and *k*_−CS_. Such slow CR is consistent with the large driving force of this process, and an electron transfer occurring in the inverted region as predicted by Marcus theory.^52^ However, according to this theory, CR should become faster upon increasing solvent polarity, because its driving force should decrease with the increased stabilisation of the CS state. Such an effect is possibly visible in the more polar mixtures, *i.e.* at 60% BCN and higher, where *k*_−CS_ is lower and the equilibrium more shifted toward the CS state. It is indeed important to note that *k*_CR_ is the rate constant with the highest uncertainty, because it is an order of magnitude smaller than *k*_LE_, *k*_CS_ and *k*_−CS_. As a consequence, its relative weight on the parameters *λ*_i_ and *A*_i_ in eqn (2) and (3) is almost negligible. Additionally, close inspection of the best-fit data and residuals (Fig. S10 and S11) reveals that the decay of the CS state population on the tens of nanoseconds timescale becomes increasingly non-exponential as the BCN content of the mixture increases. This behaviour could be due to electron and/or hole hopping between the three PDI units of Cage,^53^ or to the decays of sub-populations of the CS state with distinct structures. Indeed, one could anticipate that the Coulomb interaction between the PDI˙^+^ and PDI˙^−^ sub-units leads to some distortion of Cage. Furthermore, the interior of Cage is large enough to accommodate solvent molecules.^14^ Given that we are using solvent mixtures, the composition of the mixture inside Cage may vary significantly from one molecule to another. For example at low BCN content, one can anticipate the existence of a subpopulation of Cage with only TOL inside. In this case, one could reasonably assume SB-CS to be energetically less favourable than for a cage containing one or more BCN molecules. This inhomogeneity in the composition of the interior of Cage could explain the difficulty to properly reproduce the TA data at 20% BCN using the Scheme in Fig. 4 where an homogeneous composition is assumed.

**Fig. 7:**
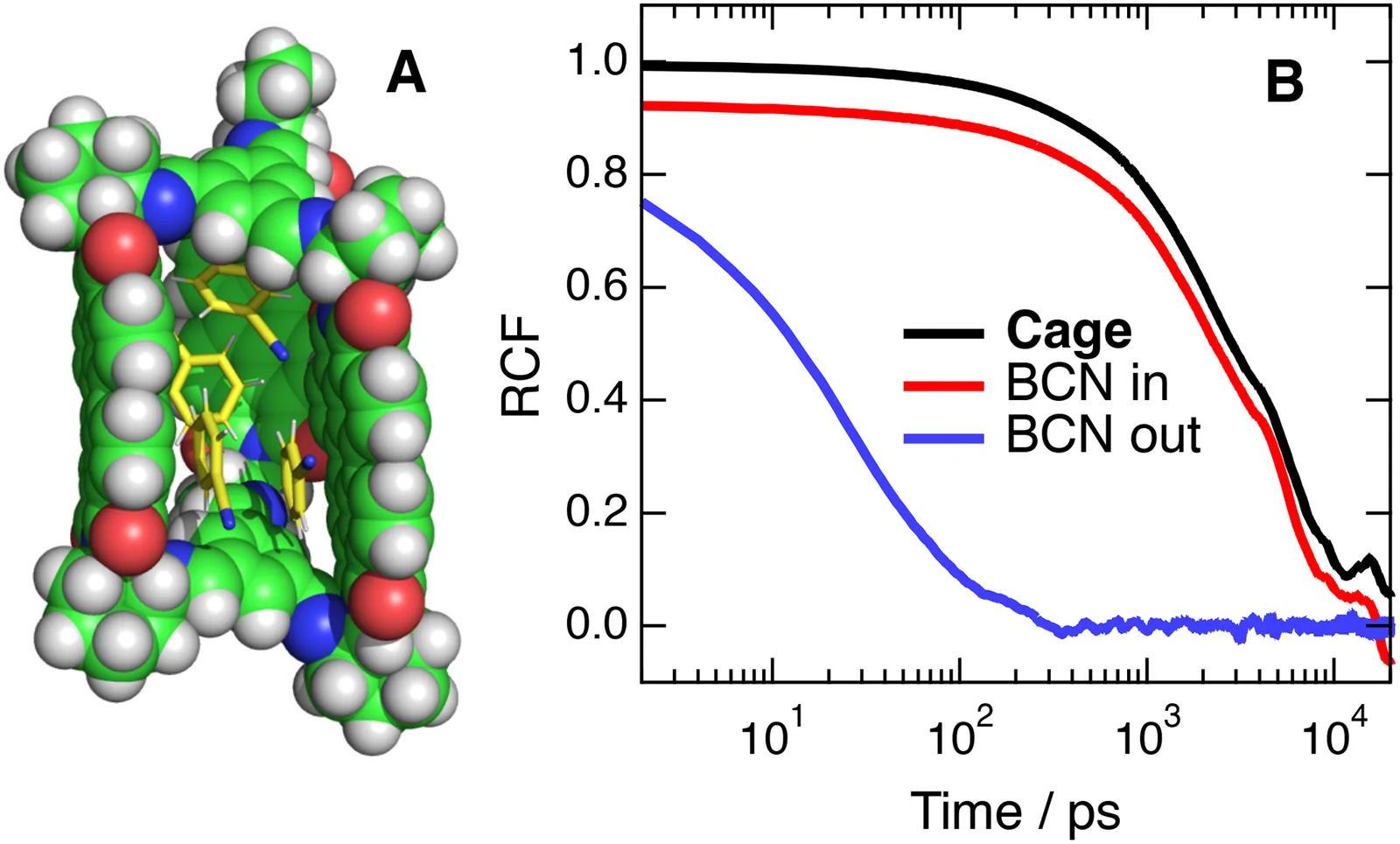
(A) Snapshot from a molecular dynamics simulation in benzonitrile (BCN) showing Cage with four solvent molecules inside. (B) Rotational correlation functions (RCF) of Cage, of a BCN molecule inside and of a BCN molecule outside calculated from the simulations. The decay time of the RCF of Cage and of a BCN inside is of the order of a few ns, and that of bulk BCN amounts to 70–80 ps.

To obtain a better insight into this question, we performed MD simulations of Cage in TOL, BCN and in a 80 : 20 (v/v) TOL/BCN mixture. The simulations in pure TOL and BCN suggest that Cage can contain between one and four solvent molecules (Fig. S19), a snapshot of Cage with four BCN inside being shown in Fig. 7A. Typically, TOL and BCN molecules leave or enter the cage on a few ns to a tens of ns timescale, respectively, suggesting that the solvents molecule are not fully trapped but that their in-and-out motion is significantly hindered by the PDI sub-units. The faster in-out exchange in TOL is consistent with the smaller viscosity of this solvent, 0.55 *vs.* 1.25 cP for BCN. The reduced mobility of these 'caged' molecules is further confirmed by their rotational correlation time similar to that of the cage itself for both solvents, and much slower than that of bulk solvent (Fig. 7B). This suggests that the orientational motion of these solvent molecules within the cage is strongly inhibited. Therefore, because of their slow response to a change of local electric field, caged BCN molecules should not contribute as much to the stabilisation of the CS state as those located outside. In other words, the effective polarity of these confined molecules should be significantly smaller than that of bulk BCN.

The MD simulations in a 80 : 20 (v/v) TOL/BCN mixture suggest that around 50% of the cages have only TOL inside, ∼37% contain one BCN and the others two or more polar solvent molecules (Fig. S20). Consequently, despite the reduced effective polarity of confined BCN, SB-CS in TOL-filled cages can be expected to be energetically less favourable than for cages containing one or more BCN, as discussed above.

Moreover, according to these simulations, the structure of Cage in solution is not as symmetric as predicted from gas-phase quantum-chemical calculations and NMR experiments,^14^ and depends on the number of solvent molecules inside. For example, to accommodate four TOL or BCN, Cage adopts a box-like shape with two PDIs parallel to each other and the third one perpendicular (Fig. 7A). The NMR experiments show clearly that these structures average on a few µs timescale and cannot be resolved with this technique. Consequently, such distributions of the content of Cage and of the mutual orientation/distance between the PDI sub-units can be expected to translate into distributions of the rate constants of the various charge-transfer processes, namely *k*_CS_, *k*_−CS_ and *k*_CR_. This effect could be anticipated to be the most pronounced at low BCN content and could explain the poorer fit of the model in Fig. 4 to the TA data discussed above. Unfortunately, including such distributions in the kinetic model would involve too many additional parameters to make a global analysis of the TA data meaningful.

With this caveat concerning the rate constants in mind, *k*_CS_ and *k*_−CS_ were used to obtain the equilibrium constant between the LE and CS states and then to estimate the SB-CS driving force, Δ*G*_SBCS_. As shown in Table 1, SB-CS in Cage is predicted to be slightly endergonic below 60% BCN content and to be weakly exergonic above. These small Δ*G*_SBCS_ values account for the observation of delayed fluorescence in solvent mixtures as well as in pure BCN. Significantly more exergonic SB-CS, *i.e.* Δ*G*_SBCS_ = −380 meV, was predicted in BCN from the Weller equation with the experimentally determined redox potentials of the PDI monomer.^14^ With such driving force, SB-CS would be essentially irreversible, and no delayed fluorescence would be observed, which is the case for the majority of PDI multichromphoric systems displaying SB-CS studied previously.^5,11,13,15,17,36,37,47,54^ This discrepancy most probably arises from the redox potentials measured with the PDI monomer and not with Cage. In addition, cyclic voltammetry captures redox behaviour of well-equilibrated populations on timescales longer than that of the NMR, where Cage appears fully symmetric. Therefore, nuances of solvent confinement affecting the solvation of Cage in the SB-CS cannot be resolved. Given the lower effective polarity of caged solvent suggested by the MD simulations, the solvation energy of the CS state in Cage should be significantly smaller than the sum of those of the PDI monomer ions. Because of this, SB-CS in Cage can be expected to be significantly less exergonic than intermolecular SB-CS between two PDIs.

## 3. Conclusions

These results illustrate the strength of combining TA and time-resolved fluorescence spectroscopies when investigating weakly exergonic SB-CS processes. Whereas the spectral signature of the CS state may be difficult to detect from the raw TA data, the presence of delayed fluorescence is an unambiguous indicator of an equilibrium between the LE state and a dark state. Additionally, combined analysis of the TA and fluorescence data allows for a more reliable determination of the various kinetic parameters. Using this approach, we were able to detect SB-CS in a PDI cage in a broad range of solvent polarity. Interestingly, this process is reversible even in highly polar media like BCN. This contrast with previous investigations on SB-CS with PDI-based systems in polar media where SB-CS was ultrafast and irreversible.^5,11,15,17,33,34,36,37^ This difference most probably originates from the presence of solvent molecules inside Cage, which have a limited mobility and thus a reduced ability to stabilise the CS state. In this respect, such 'porous' molecular architectures could allow for a fine tuning of photoinduced charge-transfer dynamics, which may prove very useful for various applications.

## Author contributions

E. S-R. performed the spectroscopic measurements, developped the data analysis model with R. J. F-T., who also carried out the quantum-chemical calculations, and wrote the first draft of the manuscript. H-H. H. synthesised the compound under the supervision of T. Š.; E. V. performed the MD simulations and wrote the final version of the manuscript with the help of all co-authors.

## Conflicts of interest

There are no conflicts to declare.

## Supplementary Material

CP-028-D6CP02527G-s001

## Data Availability

All data can be downloaded from https://doi.org/10.26037/yareta:maqum7obdndezdcp7aih7747p4. Supplementary information (SI): experimental details, description of the model and of the data analysis, stationary electronic spectroscopy, time-resolved fluorescence, transient absorption data, quantum-chemical calculations, and MD simulations. See DOI: https://doi.org/10.1039/d6cp02527g.
