# Supplementary material for: Detecting photoinduced symmetry-breaking charge separation with near-zero driving force in a perylene diimide cage
Source: Phys Chem Chem Phys. 2026 Aug 3. Online ahead of print. doi: 10.1039/d6cp02527g (PMC13430495; doi:10.1039/d6cp02527g)
Supplement: CP-OLF-D6CP02527G-s001 [file CP-OLF-D6CP02527G-s001.pdf]

Supplementary Information (SI) for Physical Chemistry Chemical Physics.
This journal is © the Owner Societies 2026

Supporting Information for:

Detecting Photoinduced Symmetry-Breaking Charge Separation with Near-Zero Driving Force in a Perylene Diimide Cage

Estefanía Sucre-Rosales,[a] Ricardo J. Fernández-Terán,[a] Hsin-Hua Huang,[b] Tomáš Šolomek,[bc]* and Eric Vauthey[a]*

[a] *Department of Physical Chemistry, University of Geneva, CH-1205 Geneva, Switzerland.*
[b] *Department of Chemistry, University of Basel, St. Johanns-Ring 19, CH-4056 Basel, Switzerland.*
[c] *Van't Hoff Institute for Molecular Sciences (HIMS), University of Amsterdam, 1090 GD Amsterdam, The Netherlands.*

**E-mail:* t.solomek@uva.nl, eric.vauthey@unige.ch
(Dated: July 24, 2026)

Contents

Page

S1 Methods **S3**
- S1.1 Solvents and reactants S3
- S1.2 Stationary electronic absorption and fluorescence S3
- S1.3 Time-correlated single photon counting S3
- S1.4 Transient absorption spectroscopy S3
  - S1.4.1 pump-probe measurements S3
  - S1.4.2 Spectral stitching of UV and NIR datasets S4
- S1.5 Quantum-chemical calculations S5
- S1.6 Molecular dynamics simulations S5

S2 Description of the model **S6**
- S2.1 Definition of the model S6
- S2.2 Generalisation to Arbitrary Number of Initial Relaxation Steps S7
- S2.3 Applying the Constraints to the Fit S8
- S2.4 Best-fit parameters S9

S3 Additional results **S10**
- S3.1 Stationary spectra S10
- S3.2 Time-resolved fluorescence S12
- S3.3 Transient absorption S13
- S3.4 Quantum chemical calculations S18
- S3.5 Molecular dynamics simulations S20

S4 References **S20**

List of Figures

Page

Fig. **S1** Target scheme used to analyse the transient absorption data recorded with **Cage**. S6
Fig. **S2** Flowchart summarizing the fitting protocol used in the present work. LM refers to the Levenberg-Marquadt algorithm. S9
Fig. **S3** Stationary absorption and emission spectra of **Cage** in toluene/benzonitrile (TOL/BCN) mixtures. S10
Fig. **S4** Structure of the commercial PDI analogue used for spectroelectrochemistry. S11
Fig. **S5** Measured (raw) and subtracted+scaled absorption spectrum of $PDI^{\bullet+}$. Lower panel: Spectral noise calculated during the acquisition S11

Fig. **S6** Time resolved fluorescence measured with **Cage** in different solvent mixtures (blue), instrument response function (gray) and best biexponential fit (red). Top: autocorrelation of the residuals (green); Bottom: normalised residuals (red). Panels b and c show that the fluorescence decay at 20% BCN is better described with a biexponential function. S12

Fig. **S7** a) Structure of the PDI **Monomer** b) Transient absorption spectra measured upon 525 nm excitation of **Monomer** in BCN S13

Fig. **S8** Evolution-associated difference spectra obtained from a global analysis of the merged femtosecond-nanosecond transient absorption data measured with **Monomer** in BCN, assuming a series of two successive exponential steps (A $\rightarrow$ B $\rightarrow$). . . S13

Fig. **S9** Species-associated difference absorption spectra obtained from a constrained target the merged femtosecond-nanosecond transient absorption spectra measured with **Cage** in various BCN/TOL mixtures. S14

Fig. **S10** Contour plots of the merged femtosecond-nanosecond transient absorption measured with **Cage** in various BCN/TOL mixtures (left), best fits of the target analysis (centre) and residuals (right). S15

Fig. **S11** Upper panel: Transient absorption at selected wavelengths (circles) measured with **Cage** in TOL/BCN mixtures and best fits (solid lines). Lower panel: Residuals. S16

Fig. **S12** a) Stationary absorption spectra of PDI$^{\bullet+}$and PDI$^{\bullet-}$ taken from ref. 1,2. b) Evolution-associated difference spectra and time constants obtained from global analysis of the merged transient absorption data measured with **Cage** in TOL assuming a series of two successive exponential steps (A $\rightarrow$ B$\rightarrow$). c,d) same as b) but in 80:20 TOL/BCN mixture assuming three successive exponential steps (A $\rightarrow$ B$\rightarrow$ C$\rightarrow$), with increasing time constants (c) or with inverted kinetics (d). e,f) Species-associated difference absorption spectra obtained from a constraint target analysis of the data measured with **Cage** in 80:20 TOL/BCN mixture (e) and pure BCN (f). S17

Fig. **S13** Structure of the PDI analogue used for calculations. S18

Fig. **S14** TD-DFT calculated electronic absorption spectra of the relevant species derived from the PDI model compound (Figure **S13**). The solid lines represent the convolved spectra (*left scale*), whilst the vertical bars indicate the oscillator strengths (*right scale*). S18

Fig. **S15** Comparison of the experimental and TD-DFT calculated electronic absoprtion spectra of the PDI model compound (Figure **S13**). The solid lines represent the convolved spectra (*left scale*), the vertical bars indicate the oscillator strengths. . . . S18

Fig. **S16** Comparison of the experimental absortion spectrum of the triplet excited state of the commercial PDI compound (Figure **S4**) in ACN measured by transient-absorption spectroscopy,[3] and of the TD-DFT calculated spectrum of the ^{3}PDI* model compound (Figure **S13**). The solid lines represent the convolved spectra (*left scale*), the vertical bars indicate the oscillator strengths. S19

Fig. **S17** Comparison of the experimental absorption spectrum of the commercial PDI$^{\bullet-}$ (Figure **S4**) in BCN measured using spectroelectrochemistry and of the TD-DFT calculated spectra of the PDI$^{\bullet-}$ model compound (Figure **S13**). The solid lines represent the convolved spectra (*left scale*), the vertical bars indicate the oscillator strengths. S19

Fig. **S18** TD-DFT calculated spectra of PDI$^{\bullet+}$ from the PDI model compound (Figure **S13**). The solid lines represent the convolved spectra (*left scale*), the vertical bars indicate the oscillator strengths, and the experimental spectrum of the commercial PDI compound (Figure **S4**) in ACN measured using spectroelectrochemistry is shown for comparison (*right scales*). S19

Fig. **S19** Histograms of the number of solvent molecules within 2.9 Å of the centre of mass (COM) of **Cage** from MD simulations in toluene (left) and benzonitrile (right). **Cage** can accommodate up to four TOL or BCN molecules entirely. Larger values in the histograms correspond to cases where some solvent molecules are not entirely located inside **Cage**. S20

Fig. **S20** Histogram of the number of benzonitrile (BCN) and toluene (TOL) molecules within 2.9 Å of the centre of mass of **Cage** from MD simulations in a 80:20 (v/v) TOL/BCN mixture. S20

S1. Methods

S1.1 Solvents and reactants

All solvents were of HPLC and/or spectroscopic grade and were used as received. The PDI **Cage** was synthesized as described in the literature.[2]

S1.2 Stationary electronic absorption and fluorescence

All samples were measured in 1 cm cuvettes. The stationary electronic absorption spectra were measured using a Cary 50 spectrometer, while the fluorescence emission spectra were recorded using a Horiba FluoroMax-4 spectrofluorimeter with 525 nm excitation.

The absorption spectra of the radical anion and cation of PDI, $PDI^{\bullet -}$ and $PDI^{\bullet +}$, were obtained by bulk electrolysis of commercial PDI (see below) at the corresponding peak anodic/cathodic in an OTTLE cell (Pt working and counter electrodes, Ag pseudo-reference electrode) in the corresponding solvent with 0.1 M [TBA][PF_6] as supporting electrolyte. The spectra were collected with a fiber-coupled spectrometer (Avantes AvaSpec ULS2048CL EVO spectrometer) using a deuterium+tungsten hybrid balanced source (Avantes AvaLight-DH-S-BAL), with short integration times (0.1 ms) and 128 spectral averages. In all cases, the electrolyte solution was used to collect the reference spectrum, and the final spectra were obtained after no further changes were observed and a negligible current flow was detected by the potentiostat.

$PDI^{\bullet -}$ was measured in benzonitrile. Attempts to obtain the spectrum of $PDI^{\bullet +}$ in benzonitrile (BCN) were unsuccessful due to the close proximity of the solvent oxidation peak, hence this was performed in acetonitrile (ACN), which has a larger electrochemical window. For $PDI^{\bullet +}$, since no quantitative conversion was possible in ACN, the neutral species spectrum was subtracted with a fitted scaling factor (0.406±0.001), imposing a non-negativity criterion in the spectral region dominated by the absorption features of the neutral PDI (Figure **S5**).

S1.3 Time-correlated single photon counting

All samples were bubbled with nitrogen flow during 15 minutes prior to measurement. The excitation was performed at a repetition rate of 40 MHz with <90 ps pulses generated by a laser diode at 469 nm (Picoquant model LDH-P-C-470). Fluorescence was collected at 90° with a polarization at magic angle to that of the pump pulses. The detected fluorescence wavelength was selected with an interference filter. A photomultiplier tube (Hamamatsu, H5783-P-01) was used as a detector, whose output was connected to the input of a TCSPC computer board module (Becker and Hickl, SPC-300- 12). The full width at half-maximum (FWHM) of the instrument response function (IRF) was around 200 ps. The accuracy on the lifetimes is of ca. 0.1 ns.

S1.4 Transient absorption spectroscopy

All samples were kept under nitrogen flow during the entire duration of the measurement (around 1.5-2 h).

S1.4.1 pump-probe measurements

fs-TA pump: Excitation with the fsTA was performed at 525 nm, with the output a TOPAS-Prime in combination with a NirUVis frequency mixer (both from Light Conversion), seeded by the output of a 5 kHz Ti:Sapphire amplified system (Spectra Physics, Solstice Ace), and chopped to 500 Hz.

fs-TA VIS probe: E The white-light continuum was generated by focusing around 1 μJ of 800 nm pulses (1 KHz) onto a moving 3 mm CaF_2 plate. Both pump and probe pulses were focused and overlapped onto the sample (100 and 60 μm diameter, respectively). The transient absorption signal was checked prior to the measurement to scale linearly with the pump intensity. The polarization of the pump pulses was set to magic angle relative to the white-light pulses. For the data treatment, the pixel-to-wavelength conversion was done using a standard filter of Holmium oxide, which shows narrow bands in the UV-Vis spectral region. A full description of the setup can be found in ref. 4.

fs-TA NIR probe: The white light was generated by focusing the 800nm pulses in a YAG crystal. To balance the white light spectrum, the intense 800nm light was removed by a beam stop after generation as well as by a 1mm cuvette containing IR140 in DMSO. The probe light was then split into a reference and a sample beam using a reflective metallic neutral density filter. After passing the sample, the beam was dispersed in a home-built prism spectrometer and the intensity recorded with a InGaAs detector. To balance the white light spectrum, apodizing neutral density filters were placed directly before both detectors. The pixel to wavelength conversion was achieved using a standard containing rare earth metals (NIST 2065 for NIR), which shows narrow bands from the UV to the NIR. A full description of the setup can be found in ref. 5.

ns-TA setup: The excitation was achieved using a passively Q-switched, frequency doubled Nd:YAG laser (Teem Photonics, Powerchip NanoUV) producing pulses at 532 nm, 500 Hz repetition rate, with approximately 20 μJ, and 300 ps duration. The UV-VIS white-light continuum was generated in an identical way as previously described. A full description of the setup used can be found in ref. 6.

S1.4.2 Spectral stitching of UV and NIR datasets

To construct broadband transient absorption spectra covering the range from 350 to 1260 nm, the independent ultraviolet/visible (UV) and near-infrared (NIR) datasets were merged along the wavelength axis. Let $\boldsymbol{S}_{\mathrm{UV}}(\lambda, t)$ and $\boldsymbol{S}_{\mathrm{NIR}}(\lambda, t)$ denote the time-resolved spectral matrices for the UV and NIR detection windows, respectively, where λ and t represents respectively the wavelength and the time delay between pump and probe pulses.

Time-axis synchronization: Prior to spectral stitching, the time axes of the two datasets were synchronized. The UV dataset was interpolated onto the delay vector of the NIR dataset using a linear interpolation scheme to ensure a one-to-one correspondence between time points t during the scaling optimization.

Scaling factor determination: A multiplicative scaling factor, α, was determined to normalize the amplitude of the UV data to match the NIR data within the spectral overlap region, defined as $\lambda \in [\lambda_{\mathrm{min}}, \lambda_{\mathrm{max}}]$ (typically 700–750 nm). To minimize the impact of pixel-to-pixel noise during scaling, we employed a polynomial surface reconstruction method. For both datasets, the spectral intensity at each time delay was approximated by a 3rd-degree polynomial, yielding smoothed matrix surfaces $\tilde{\boldsymbol{S}}_{\mathrm{UV}}(\lambda, t)$ and $\tilde{\boldsymbol{S}}_{\mathrm{NIR}}(\lambda, t)$.

The scaling factor α was obtained by minimizing the sum of squared residuals over the valid time points ($t > -0.1$ ps) and the overlap wavelength region:

$$\alpha = \underset{C}{argmin} \sum_{t} \sum_{\lambda} \left[C \cdot \tilde{\boldsymbol{S}}_{\mathrm{UV}}(\lambda, t) - \tilde{\boldsymbol{S}}_{\mathrm{NIR}}(\lambda, t) \right]^2 \tag{S1}$$

where C is the optimization variable representing the candidate scaling factor.

Construction of the merged matrix: The final merged dataset, $\boldsymbol{S}_{\mathrm{merged}}(\lambda, t)$, was constructed via concatenation at a defined cut-off wavelength, λ_{cut} (typically 730 nm), to avoid interpolation artifacts in the spectral domain. The scaled UV data were retained for wavelengths shorter than the cutoff ($\lambda < \lambda_{\mathrm{cut}}$), and the raw NIR data were retained for wavelengths equal to or longer than the cutoff ($\lambda \geq \lambda_{\mathrm{cut}}$):

$$\boldsymbol{S}_{\mathrm{merged}}(\lambda, t) = \begin{cases} \alpha \cdot \boldsymbol{S}_{\mathrm{UV}}(\lambda, t) & \text{for } \lambda < \lambda_{\mathrm{cut}} \\ \boldsymbol{S}_{\mathrm{NIR}}(\lambda, t) & \text{for } \lambda \geq \lambda_{\mathrm{cut}} \end{cases} \tag{S2}$$

Any wavelength channels containing missing values (NaN) or saturation artifacts were excluded prior to the merging process.

Temporal Merging of femtosecond and nanosecond datasets:

To span the full dynamic range from sub-picoseconds to microseconds, the femtosecond ($\boldsymbol{S}_{\mathrm{fs}}$) and nanosecond ($\boldsymbol{S}_{\mathrm{ns}}$) datasets were merged along the time axis. The merging procedure accounted for differences in spectral calibration, amplitude scaling, and temporal sampling rates.

Spectral calibration and alignment: To correct for minor calibration offsets between the two detection systems, a linear wavelength transformation was applied to the nanosecond dataset. The time-averaged spectral vectors, $\bar{\boldsymbol{S}}_{\mathrm{fs}}(\lambda)$ and $\bar{\boldsymbol{S}}_{\mathrm{ns}}(\lambda)$, were calculated over the temporal overlap window $t \in [0.5, 2.0]$ ns. The optimal transformation parameters, consisting of a slope m and an intercept b, were determined by minimizing the difference between the normalized spectral shapes:

$$(m, b) = \underset{m', b'}{argmin} \sum_{\lambda} \left[\hat{\boldsymbol{S}}_{\mathrm{fs}}(\lambda) - \hat{\boldsymbol{S}}_{\mathrm{ns}}(m'\lambda + b') \right]^2 \tag{S3}$$

where $\hat{\boldsymbol{S}}$ denotes the locally min-max normalized spectrum, and m' and b' are the optimization variables for slope and intercept, respectively. Spectral regions containing high-intensity artifacts (e.g., scatter at $\lambda \approx 532$ nm) were excluded from the optimization cost function to ensure robust alignment. The nanosecond dataset was subsequently interpolated onto the calibrated femtosecond wavelength axis using shape-preserving piecewise cubic interpolation (PCHIP).

Amplitude scaling: To account for differences in spectral sensitivity between the femtosecond and nanosecond detection setups, a wavelength-dependent scaling function, $\beta(\lambda)$, was calculated to match the signal amplitudes in the temporal overlap window. Kinetic traces for each wavelength were modeled using 3rd-degree polynomials along the time axis, denoted as matrices $\boldsymbol{P}_{\mathrm{fs}}(\lambda, t)$ and $\boldsymbol{P}_{\mathrm{ns}}(\lambda, t)$. The calibration function $\beta(\lambda)$ was modeled as a 2nd-order polynomial in wavelength, $\beta(\lambda) = a\lambda^2 + b\lambda + c$, and its coefficients were determined via global least-squares optimization:

$$\beta(\lambda) = \underset{\beta'(\lambda)}{argmin} \sum_{\lambda \in \Lambda_{\mathrm{sig}}} \sum_{t=0.5}^{2.0} \left[\beta'(\lambda) \cdot \boldsymbol{P}_{\mathrm{fs}}(\lambda, t) - \boldsymbol{P}_{\mathrm{ns}}(\lambda, t) \right]^2 \tag{S4}$$

To ensure a robust fit, only wavelength channels exhibiting significant amplitude (exceeding 5% of the global maximum) were included in the optimization set Λ_{sig}. The resulting function $\beta(\lambda)$ was then applied to the femtosecond dataset across the entire spectral range.

Weighted temporal blending: The final time axis was constructed by determining the union of unique time points from both datasets. The merged data matrix, $\boldsymbol{S}_{\text{final}}(\lambda, t)$, was computed using a weighted linear cross-fade function, $w(t)$, over a transition window defined by $t \in [t_{\text{start}}, t_{\text{end}}]$ (typically 0.8–1.5 ns):

$$\boldsymbol{S}_{\text{final}}(\lambda, t) = w(t) \cdot \boldsymbol{S}'_{\text{fs}}(\lambda, t) + (1 - w(t)) \cdot \boldsymbol{S}'_{\text{ns}}(\lambda, t) \tag{S5}$$

where $\boldsymbol{S}'_{\text{fs}}$ and $\boldsymbol{S}'_{\text{ns}}$ denote the datasets interpolated onto the common time axis. The weighting function $w(t)$ determines the contribution of the femtosecond dataset and is defined as:

$$w(t) = \begin{cases} 1 & \text{if } t \leq t_{\text{start}} \\ \frac{t_{\text{end}} - t}{t_{\text{end}} - t_{\text{start}}} & \text{if } t_{\text{start}} < t < t_{\text{end}} \\ 0 & \text{if } t \geq t_{\text{end}} \end{cases} \tag{S6}$$

This approach prioritizes the higher time-resolution $\boldsymbol{S}_{\text{fs}}$ data at early delays ($t \leq t_{\text{start}}$) while smoothly transitioning to the $\boldsymbol{S}_{\text{ns}}$ data for long time delays ($t \geq t_{\text{end}}$), preventing step discontinuities in the kinetic traces.

S1.5 Quantum-chemical calculations

Quantum-chemical calculation were performed at the the density functional theory (DFT) level using Gaussian 16, rev. C.02.[7] The molecules were optimised at the CAM-B3LYP/def2-SVP level of theory,[8,9] with a DMF polarisable continuum model (IEF-PCM), including Grimme's D3 dispersion with Becke–Johnson damping (`dispersion=GD3BJ`).[10] The structures of the neutral, anionic, dianionic and cationic states of a PDI analogue (Fig. **S7**) were optimised to a minimum, which was verified by the lack of imaginary frequencies in the vibrational analysis. The electronic absorption spectra were calculated using the same geometries and at the same level of theory, with time-dependent (TD) DFT considering the lowest 30 excited states. The spectra were obtained by convolution with a Gaussian function with 1500 cm^{-1} FWHM. To improve the agreement with the experimental spectra, all transitions were shifted by -2450 cm^{-1}. TD-DFT calculations of the triplet ($T_n \leftarrow T_1$) absorption spectrum (i.e. $^3\text{PDI}^*$) were performed using the optimised triplet geometry, with the initial ground-state geometry and reference orbitals, at the same level of theory as described before.

S1.6 Molecular dynamics simulations

Molecular dynamics (MD) simulations were carried out using GROMACS 2023.1.[11] The optimised structure of**Cage** was determined from quantum-chemical calculations in the gas phase at the DFT level (B3LYP/6-31G(d,p)) using Gaussian 16.[7] The topologies for **Cage** was generated using the Antechamber Python parser interface (ACPYPE),[12] with the general Amber force field (GAFF).[13] The atomic charges were determined from CHELPG fits of the electrostatic potential obtained from the quantum-chemical calculations.[14] The GAFF-ESP-2018 force field was used for the solvents.[15] A periodic cubic box (6x6x6 nm^3) was used for the simulations, which were performed at constant pressure (1 atm) and temperature (295 K) with 2 fs steps for 80 ns. The box was filled with one **Cage** and either 1100 molecules of toluene, 1100 molecules of benzonitrile, or 880 toluene and 220 benzonitrile molecules.

Non-bonded interactions were evaluated with a cutoff of 1.2 nm, and long-range electrostatic interactions were accounted for by the particle mesh Ewald method,[16] with 0.16 nm grid spacing and forth-order interpolation. A long-range dispersion correction for energy was also included. The LINCS algorithm[17] was used to constrain the bonds of all system components. The equilibration of the system was ensured by inspecting the total energy drift. The isothermal-isobaric ensemble, NPT, was used for all productions with the Noose-Hoover thermostat at 295 K,[18] and the c-rescale barostat[19] at 1 atm using coupling constants 0.5 and 5 ps respectively.

S2. Description of the model

S2.1 Definition of the model

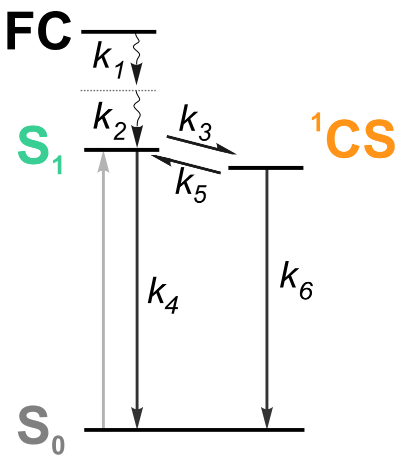

Figure S1: Target scheme used to analyse the transient absorption data recorded with **Cage**.

In the following, we shall solve the kinetic problem defined by eq. S7:

$$\frac{d}{dt}\vec{C}(t) = \boldsymbol{K} \cdot \vec{C}(t) \tag{S7}$$

It can be shown that if $\vec{C}(0)$ is a vector containing the initial populations (at $t = 0$), and if $\boldsymbol{K}$ is diagonalisable, then the general solution is given by eq. S8:[20,21]

$$\vec{C}(t) = \boldsymbol{V} e^{\boldsymbol{\Lambda} t} \boldsymbol{V}^{-1} \vec{C}(0) \ , \tag{S8}$$

where $\boldsymbol{\Lambda} \equiv \mathrm{diag}(\lambda_i)$ is a diagonal matrix containing the eigenvalues of $\boldsymbol{K}$, and $\boldsymbol{V}$ is a matrix containing the eigenvectors of $\boldsymbol{K}$ as column vectors. Since $\boldsymbol{\Lambda}$ is diagonal, then $e^{\boldsymbol{\Lambda} t} \equiv \mathrm{diag}(e^{\lambda_i t})$.

The kinetic scheme presented in Figure 1B can be expanded to include a precursor relaxation cascade (FC_1 →FC_2 →S_1) (Figure **S1**), which can be described by a 4×4 $\boldsymbol{K}$ matrix of the form ($k_i \geq 0, \ \forall i$):

$$\boldsymbol{K} = \begin{pmatrix} -k_1 & 0 & 0 & 0 \\ k_1 & -k_2 & 0 & 0 \\ 0 & k_2 & -k_3 - k_4 & k_5 \\ 0 & 0 & k_3 & -k_5 - k_6 \end{pmatrix} \tag{S9}$$

It is worth noting that $\boldsymbol{K}$ has a lower block-triangular structure. Because of this, its eigenvalues are simply the union of the eigenvalues of its diagonal elements (the sequential relaxation steps) and its lower right 2×2 block (the $S_1 \rightleftharpoons$ CS equilibration). Under these conditions, the eigenvalues of $\boldsymbol{K}$ are:

$$\lambda_1 = -k_1 \tag{S10a}$$
$$\lambda_2 = -k_2 \tag{S10b}$$
$$\lambda_3 = -\frac{1}{2}\left(\Omega + \sqrt{\Omega^2 - 4\xi}\right) \tag{S10c}$$
$$\lambda_4 = -\frac{1}{2}\left(\Omega - \sqrt{\Omega^2 - 4\xi}\right) \ , \tag{S10d}$$

with $\Omega \equiv k_3 + k_4 + k_5 + k_6$, and $\xi \equiv k_4(k_5 + k_6) + k_3 k_6$. If we assume relaxation from the initially populated Franck–Condon states is ultrafast, we have $k_1, k_2 \gg k_{i>2}$, meaning $\lambda_1, \lambda_2 \ll \lambda_3 < \lambda_4 \leq 0$.

Since only the population in the relaxed S_1 state is emissive, the time-dependent fluorescence decay (e.g. TCSPC) is well-represented by the population kinetics of S_1, defined as a generalised sum of exponentials:

$$I(t) = \left[\sum_{i=1}^{N} a_i e^{\lambda_i t}\right] * \mathrm{IRF} \ , \tag{S11}$$

where $N = 4$ for this specific expanded scheme, $*$ denotes convolution with the instrument response function (IRF), a_i are the amplitudes, and $\lambda_i = -1/\tau_i$ are the corresponding rate constants (lifetimes). Since the transient absorption data and TCSPC data are intrinsically measured in different time units, uniformity in these must be ensured by a suitable transformation of the rate constants/lifetimes to a common base. In our case, all rate constants have been converted to $(\text{ns})^{-1}$.

In the following treatment, we will neglect the contributions from the first two terms in the summation ($a_1 e^{\lambda_1 t}$ and $a_2 e^{\lambda_2 t}$), since we believe that the $FC_1 \rightarrow FC_2 \rightarrow S_1$ cascade is complete within the IRF of our TCSPC setup (ca. 250 ps). This is equivalent to taking the limit $k_1, k_2 \to \infty$ (or equivalently, $\lambda_1, \lambda_2 \to -\infty$). Because the initial time constants only append independent eigenvalues without altering λ_3 and λ_4, taking this limit instantly vanishes the initial exponential terms for $t > 0$. The effective initial condition for the slower dynamics reduces to the instantaneous arrival of population into S_1, simplifying the non-vanishing amplitudes to:

$$a_3' = \frac{\lambda_3 + k_5 + k_6}{\lambda_3 - \lambda_4} \tag{S12a}$$

$$a_4' = \frac{-(\lambda_4 + k_5 + k_6)}{\lambda_3 - \lambda_4} , \tag{S12b}$$

where the primed amplitudes indicate that these correspond to the $k_1, k_2 \to \infty$ limit. Under these conditions, we have $a_3' + a_4' = 1$. Note that in the main text, the following variables were changed ($\lambda_3 = \lambda_p, \lambda_4 = \lambda_d, a_3' = a_p$, and $a_4' = a_d$) for readability.

Similarly, we can derive the transient population of the CS state under this same ultrafast limit ($k_1, k_2 \to \infty$). Because the system reduces to an effective initial condition where all population is instantaneously in S_1 at $t > 0$ (i.e., $[S_1]_0 = 1$ and $[CS]_0 = 0$), the time evolution of the CS state, $[CS]_t$, will be a bi-exponential function governed by the same eigenvalues λ_3 and λ_4:

$$[CS]_t = b_3' e^{\lambda_3 t} + b_4' e^{\lambda_4 t} . \tag{S13}$$

Given the initial condition $[CS]_0 = 0$, it follows that $b_4' = -b_3'$. Furthermore, the initial rate of formation of the CS state is dictated solely by the forward transfer rate from S_1:

$$\left.\frac{d[CS]_t}{dt}\right|_{t=0} = k_3 [S_1]_0 = k_3 \tag{S14}$$

Taking the derivative of eq. S13 at $t = 0$ and equating it to eq. S14 yields $b_3'(\lambda_3 - \lambda_4) = k_3$. Thus, the transient population of the CS state simplifies to:

$$[CS]_t = \frac{k_3}{\lambda_3 - \lambda_4} \left(e^{\lambda_3 t} - e^{\lambda_4 t} \right) . \tag{S15}$$

S2.2 Generalisation to Arbitrary Number of Initial Relaxation Steps

We can seamlessly generalise this concept to an arbitrary number of N sequential relaxation steps $FC_1 \rightarrow \ldots \rightarrow FC_N \rightarrow S_1$ preceding the $S_1 \rightleftharpoons {}^1CS$ equilibration. The rate matrix $\boldsymbol{K}$ becomes an $(N + 2) \times (N + 2)$ lower block triangular matrix:

$$\boldsymbol{K} = \begin{pmatrix} \boldsymbol{K}_{FC} & \mathbf{0} \\ \boldsymbol{K}_{tr} & \boldsymbol{K}_{S_1} \end{pmatrix} \tag{S16}$$

where $\boldsymbol{K}_{FC}$ is an $N \times N$ lower bidiagonal matrix containing the fast sequential relaxation rates k_j $(j = 1, \ldots, N)$, $\boldsymbol{K}_{S_1}$ is the 2×2 matrix describing the slow $S_1 \rightleftharpoons {}^1CS$ system (governed by internal rates k_{N+1} through k_{N+4}), and $\boldsymbol{K}_{tr}$ couples the final Franck–Condon state to S_1. The eigenvalues of this system are simply the N relaxation rates $\lambda_j = -k_j$ (for $j = 1, \ldots, N$) and the two roots $\lambda_{N+1}, \lambda_{N+2}$ originating from $\boldsymbol{K}_{S_1}$, which are mathematically identical to λ_3 and λ_4 defined in eq. S10.

Because of this block structure, it does not matter how many ultrafast time constants N we include at the beginning; the dynamics of the slow $S_1 \rightleftharpoons {}^1CS$ subsystem remain rigorously decoupled from the upstream kinetics. When taking the limit $k_j \to \infty$ $(\forall j \leq N)$, all $a_j e^{\lambda_j t}$ terms vanish instantly in the time-resolved fluorescence. In the ultrafast transient absorption data, the initial relaxation steps may manifest as shifts or changes in the line shapes of the observed spectral features, which deviate from the time-invariance of the spectra assumed in classical kinetics. These features are often approximated by a series of consecutive exponential steps, despite their time evolution often being non-exponential in nature.[22] For our purposes herein, we have approximated this relaxation by two sequential steps.

In any case, under these assumptions, the effective initial condition always reduces to an instantaneous unit population in S_1 at $t > 0$ for TCSPC, guaranteeing that the amplitude equations for the observable S_1 decay (eq. S12) and the ${}^1CS$ transient population (eq. S15) remain valid regardless of the length of the precursor cascade, thus providing a robust set of constraints to combine time-resolved absorption and emission measurements.

S2.3 Applying the Constraints to the Fit

Due to the large number of free parameters in the fit, we sought to implement a series of physically meaningful constraints. For the optimisation problem, we used the `fmincon` minimisation routine, as implemented in MATLAB R2022a. In our implementation, we find the set of parameters $\hat{\beta}$ which minimises a function $f(\beta)$ subject to constraints. The `fmincon` solver attempts to find the optimum parameter vector ($\hat{\beta}$) which satisfies:

$$\min_{\beta} f(\beta) \quad \text{such that} \begin{cases} c(\beta) \leq 0 & \text{(S17a)} \\ c_{\text{eq}}(\beta) = 0 & \text{(S17b)} \\ A \cdot \beta \leq b & \text{(S17c)} \\ A_{\text{eq}} \cdot \beta = b_{\text{eq}} & \text{(S17d)} \\ \text{lb} \leq \beta \leq \text{ub} & \text{(S17e)} \end{cases}$$

The function to minimise consisted on the square of the norm of the residuals:[20]

$$f(\beta) = \|\boldsymbol{D}_{\text{fit}}(t, \lambda; \beta) - \boldsymbol{D}_{\text{exp}}(t, \lambda)\|^2 \tag{S18}$$

In our present case, we used the equality constraints (eq. S17b) to enforce the equalities between the fitted parameters of the TCSPC decays (two amplitudes and two lifetimes), and the two eigenvalue equations (eq. S10c and eq. S10d), plus the two amplitude equations (eq. S12a and eq. S12b), for a total of four constraints.

The options used for the `fmincon` solver were set as detailed in Table **S1**

TABLE **S1**: `fmincon` Configuration Parameters

Option	Value
`ConstraintTolerance`	10^{-3}
`FiniteDifferenceType`	`central`
`MaxFunctionEvaluations`	15000
`MaxIterations`	350
`OptimalityTolerance`	10^{-15}
`StepTolerance`	10^{-15}
`Display`	off
`Algorithm`	`sqp`
`FunctionTolerance`	10^{-15}

Furthermore, we imposed the constraint that all rate constants/lifetimes must be positive ($k_i \geq 0$ for the lower bound), and in certain cases suitable upper bounds were also defined (eq. S17e).

First, we performed a preliminary sequential analysis (A $\rightarrow$ B $\rightarrow$ C $\rightarrow$) of the data using the Levenberg–Marquardt algorithm on the fs–ns data to find k_1 and k_2. Then, the `fmincon` algorithm and the above-mentioned constraints were used in the merged fs–μs data to obtain the final rate constants, fixing k_1 and k_2 to the values obtained before (Table **S2**). We finally used the time constants obtained from this optimisation routine to get the complete species associated spectra (SAS) from the merged UV-Vis-NIR data (Figure 3) for **Cage** in the TOL/BCN solvent mixtures. The following flow chart summarizes this analysis protocol:

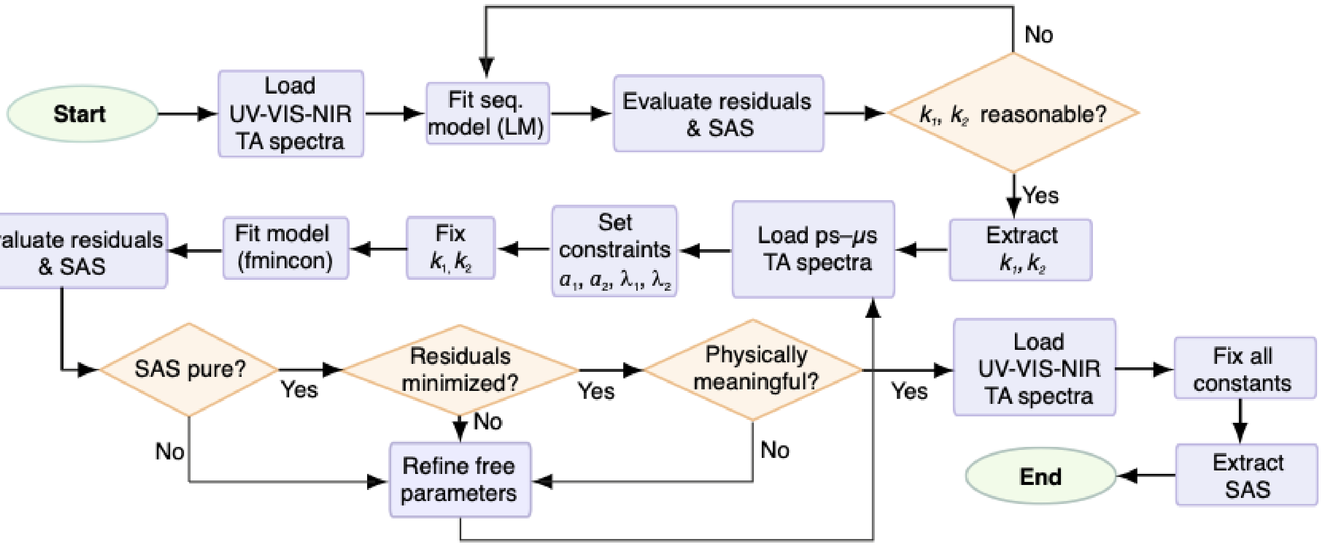

Figure S2: Flowchart summarizing the fitting protocol used in the present work. LM refers to the Levenberg-Marquadt algorithm.

S2.4 Best-fit parameters

TABLE **S2**: Best-fit parameters obtained from the contrained target analysis of the transient absorption data recorded with **Cage** in toluene(TOL)/benzonitrile(BCN) mixtures of various compositions.

%BCN[a]	τ_p[b]	τ_d	A_p	τ_1[c] (ps)	τ_2 (ps)	τ_3 (ns)	τ_4[d] (ns)	τ_5 (ns)	τ_6 (ns)
0[e]	5.46			10.8			7.2		
20	3.84	6.86	0.30	0.97	40.57	17.61	6.38	3.20	65.02
40	1.79	8.06	0.35	0.15	6.12	9.03	6.11	2.59	44.29
60	1.68	10.73	0.66	0.48	84.00	4.05	5.54	4.37	30.00
80	1.47	12.12	0.78	0.26	187.94	3.14	4.32	5.29	44.54
100	1.69	28.50	0.90	0.89	60.10	2.83	5.46	12.66	85.22

[a] %BCN benzonitrile volume content in the toluene/benzonitrile solvent mixture.
[b] τ_p and τ_d are respectively the decay time constants of prompt and delayed fluorescence, i.e. $\tau_i = 1/\lambda_i$.
[c] $\tau_1 = \tau_{\mathrm{R1}}$; $\tau_2 = \tau_{\mathrm{R2}}$; $\tau_3 = \tau_{\mathrm{CS}}$; $\tau_4 = \tau_{\mathrm{LE}}$; $\tau_5 = \tau_{-\mathrm{CS}}$; $\tau_6 = \tau_{\mathrm{CR}}$.
[d] $\tau_4 = \tau_{\mathrm{LE}}$ was constrained between 5 and 8 ns.
[e] The 0% BCN data were analysed using a series of two successive exponential steps.

S3. Additional results

S3.1 Stationary spectra

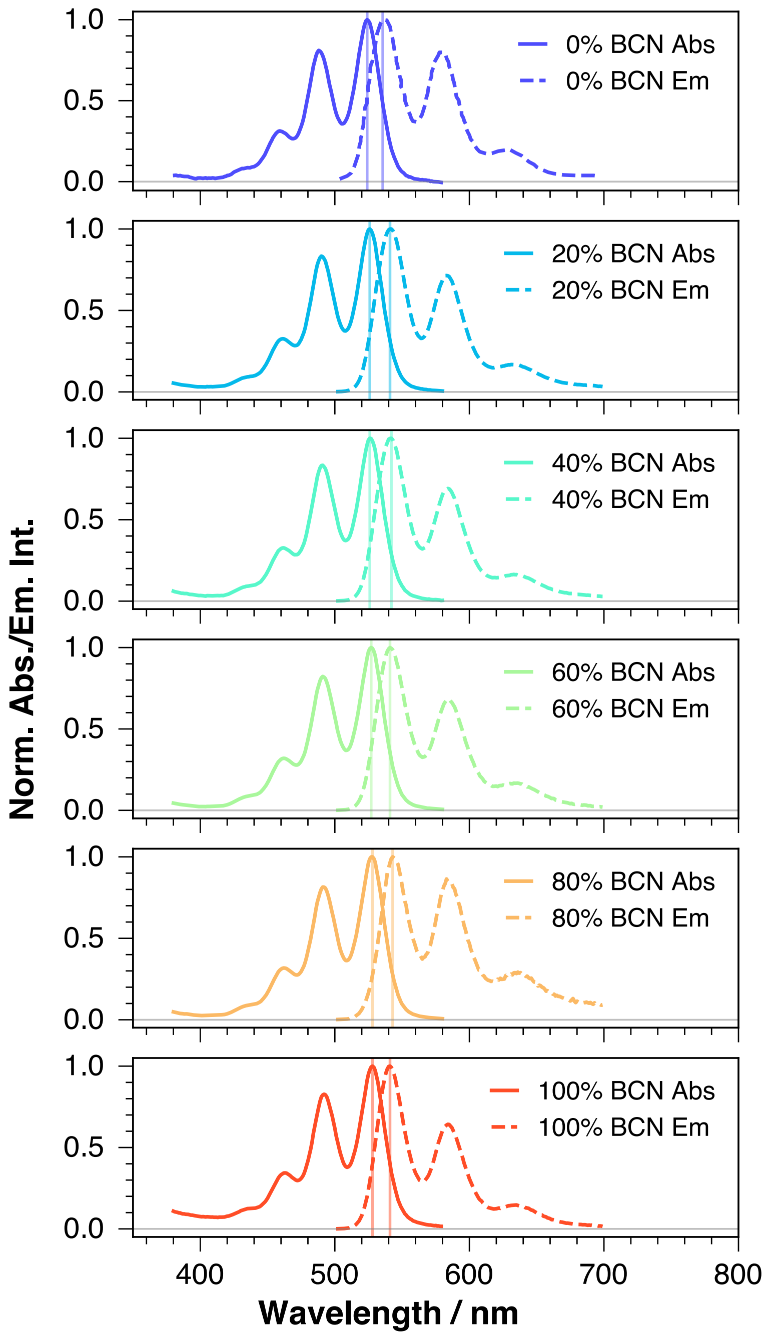

Figure S3: Stationary absorption and emission spectra of **Cage** in toluene/benzonitrile (TOL/BCN) mixtures.

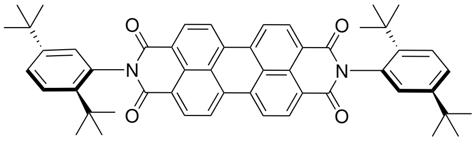

Figure S4: Structure of the commercial PDI analogue used for spectroelectrochemistry.

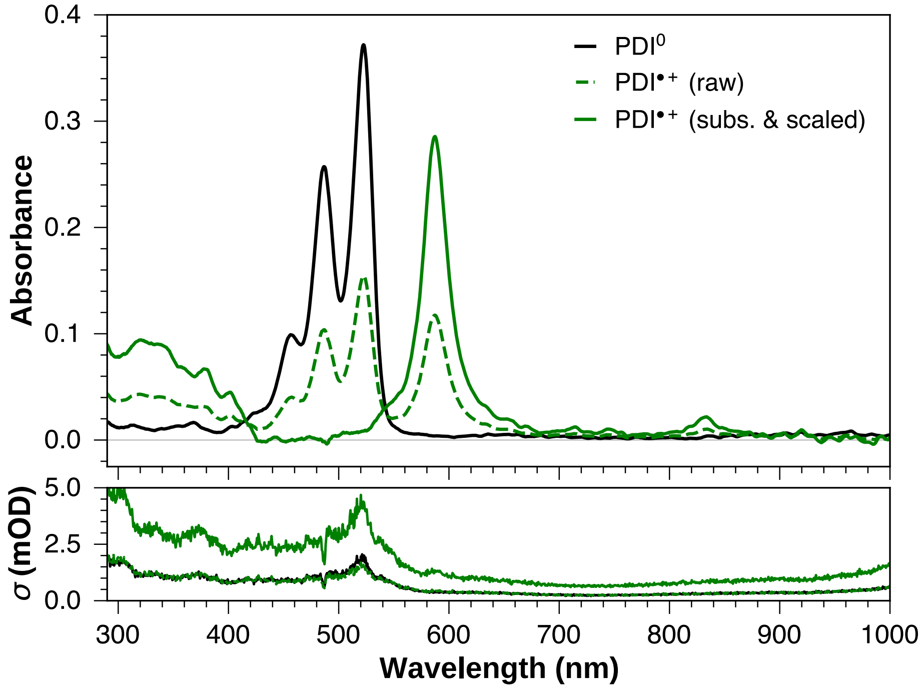

Figure S5: Measured (raw) and subtracted+scaled absorption spectrum of PDI$^{•+}$. Lower panel: Spectral noise calculated during the acquisition

S3.2 Time-resolved fluorescence

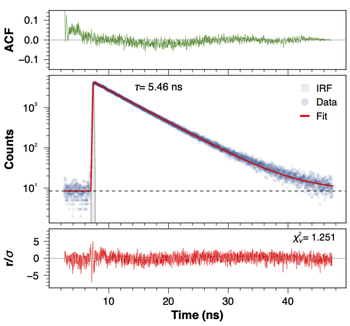

(a) TOL

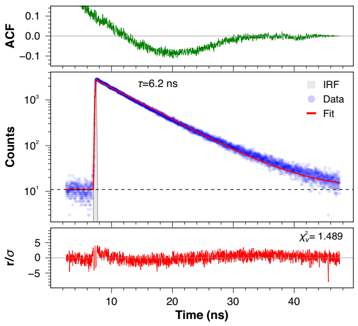

(b) 20% BCN, monoexponential

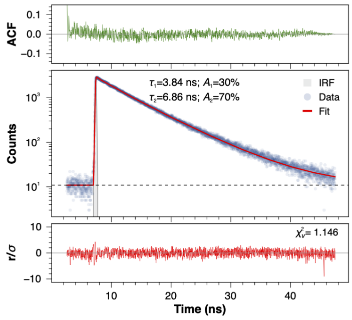

(c) 20% BCN, biexponential

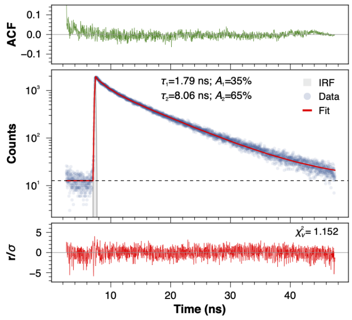

(d) 40% BCN

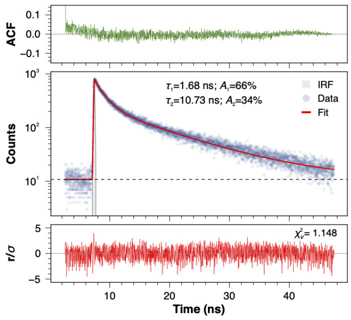

(e) 60% BCN

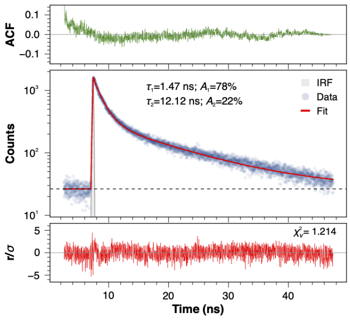

(f) 80% BCN

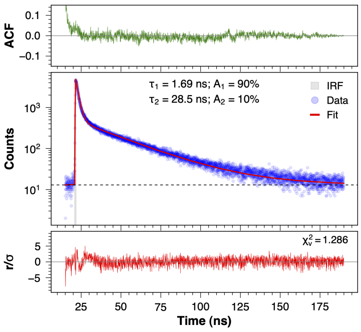

(g) BCN

Figure S6: Time resolved fluorescence measured with **Cage** in different solvent mixtures (blue), instrument response function (gray) and best biexponential fit (red). Top: autocorrelation of the residuals (green); Bottom: normalised residuals (red). Panels b and c show that the fluorescence decay at 20% BCN is better described with a biexponential function.

S3.3 Transient absorption

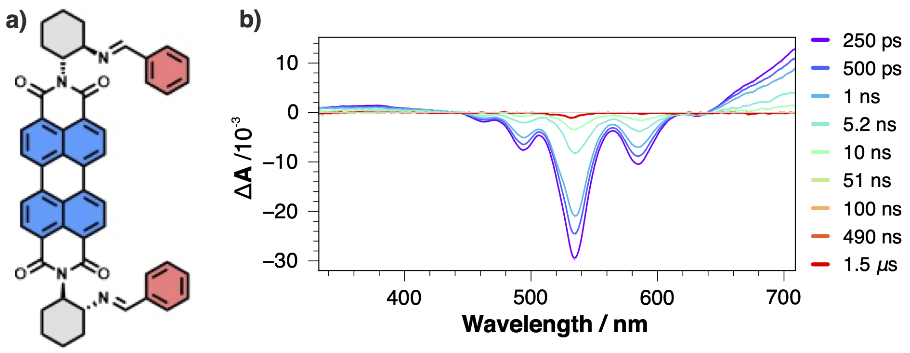

Figure S7: a) Structure of the PDI **Monomer** b) Transient absorption spectra measured upon 525 nm excitation of **Monomer** in BCN

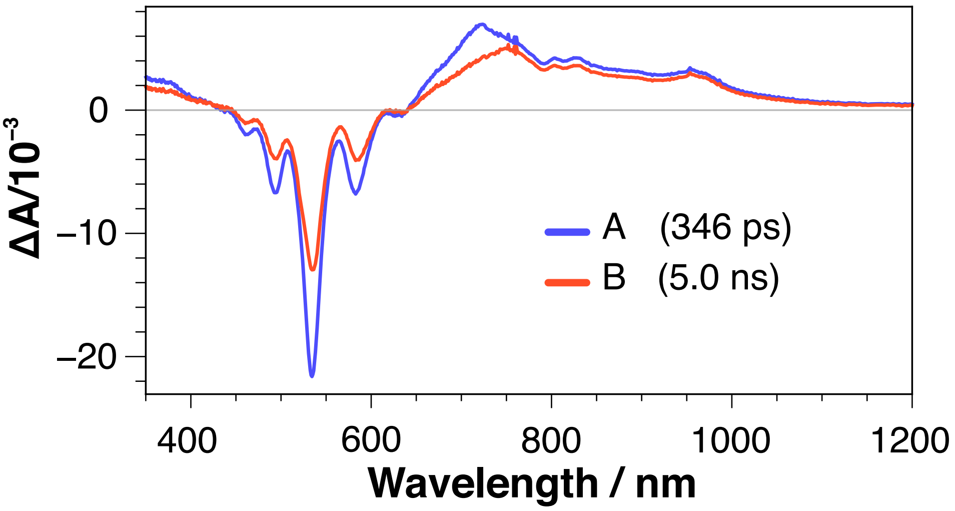

Figure S8: Evolution-associated difference spectra obtained from a global analysis of the merged femtosecond-nanosecond transient absorption data measured with **Monomer** in BCN, assuming a series of two successive exponential steps (A → B →).

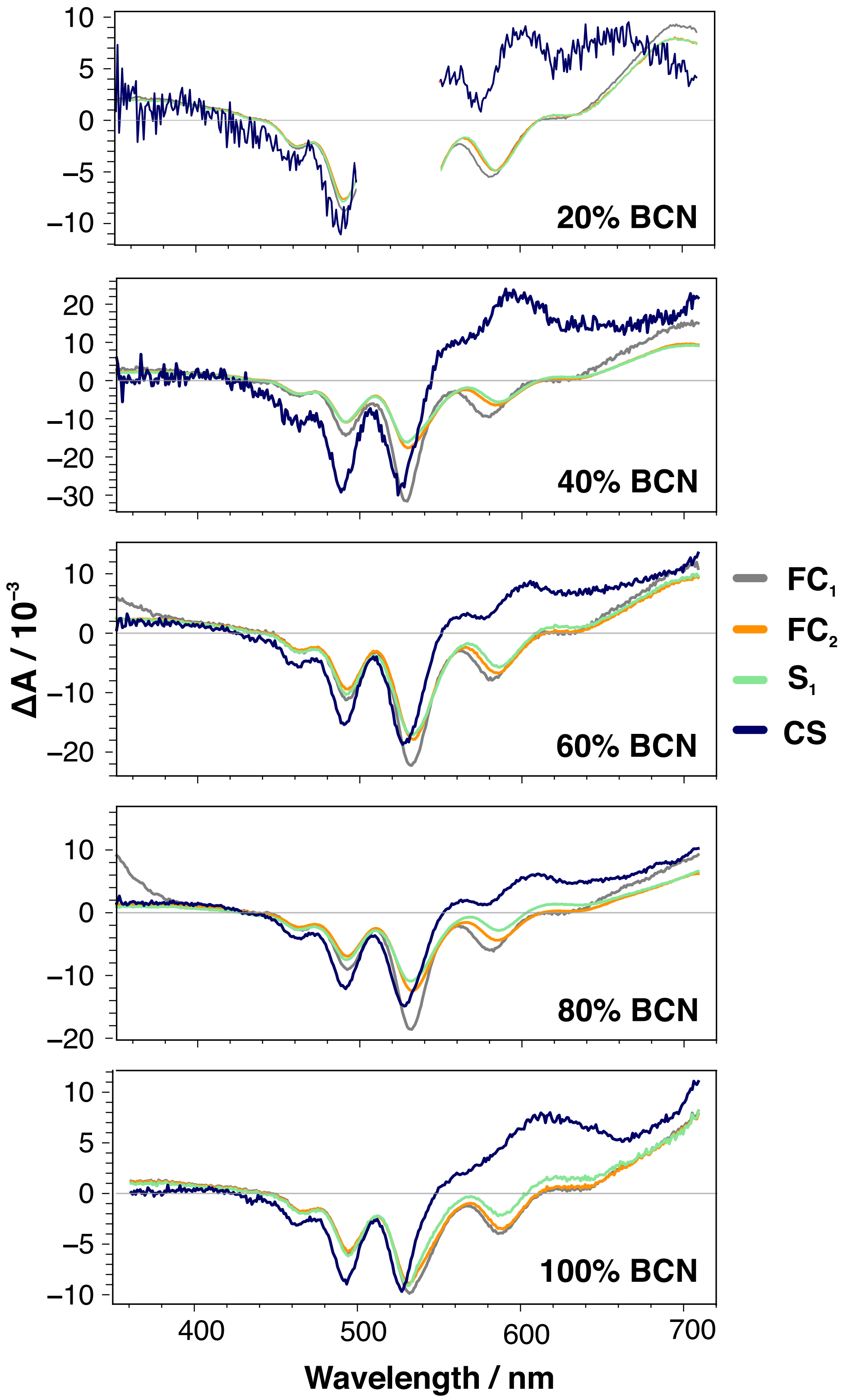

Figure S9: Species-associated difference absorption spectra obtained from a constrained target the merged femtosecond-nanosecond transient absorption spectra measured with **Cage** in various BCN/TOL mixtures.

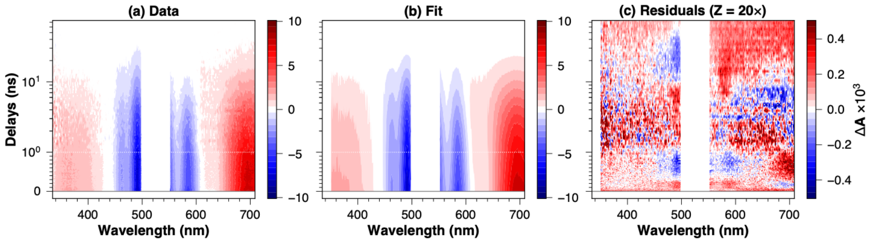

(a) 20% BCN

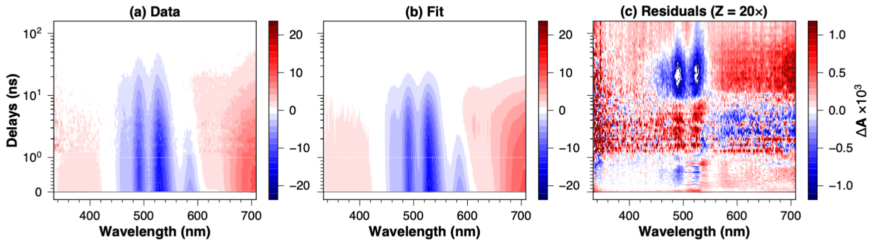

(b) 40% BCN

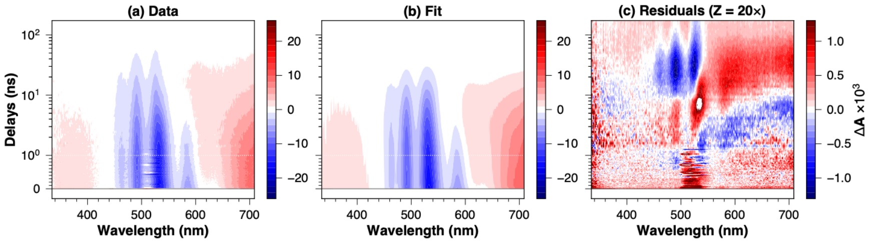

(c) 60% BCN

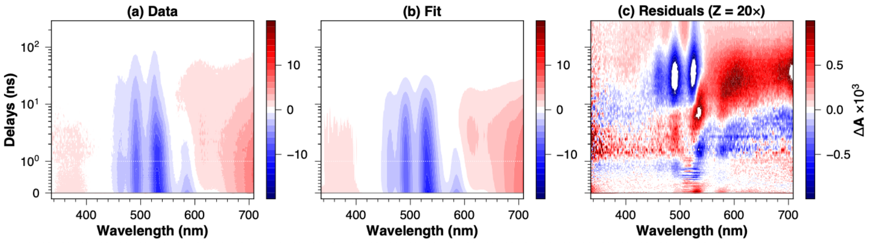

(d) 80% BCN

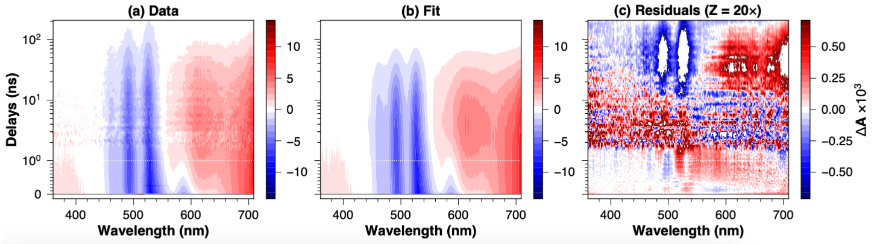

(e) BCN

Figure S10: Contour plots of the merged femtosecond-nanosecond transient absorption measured with **Cage** in various BCN/TOL mixtures (left), best fits of the target analysis (centre) and residuals (right).

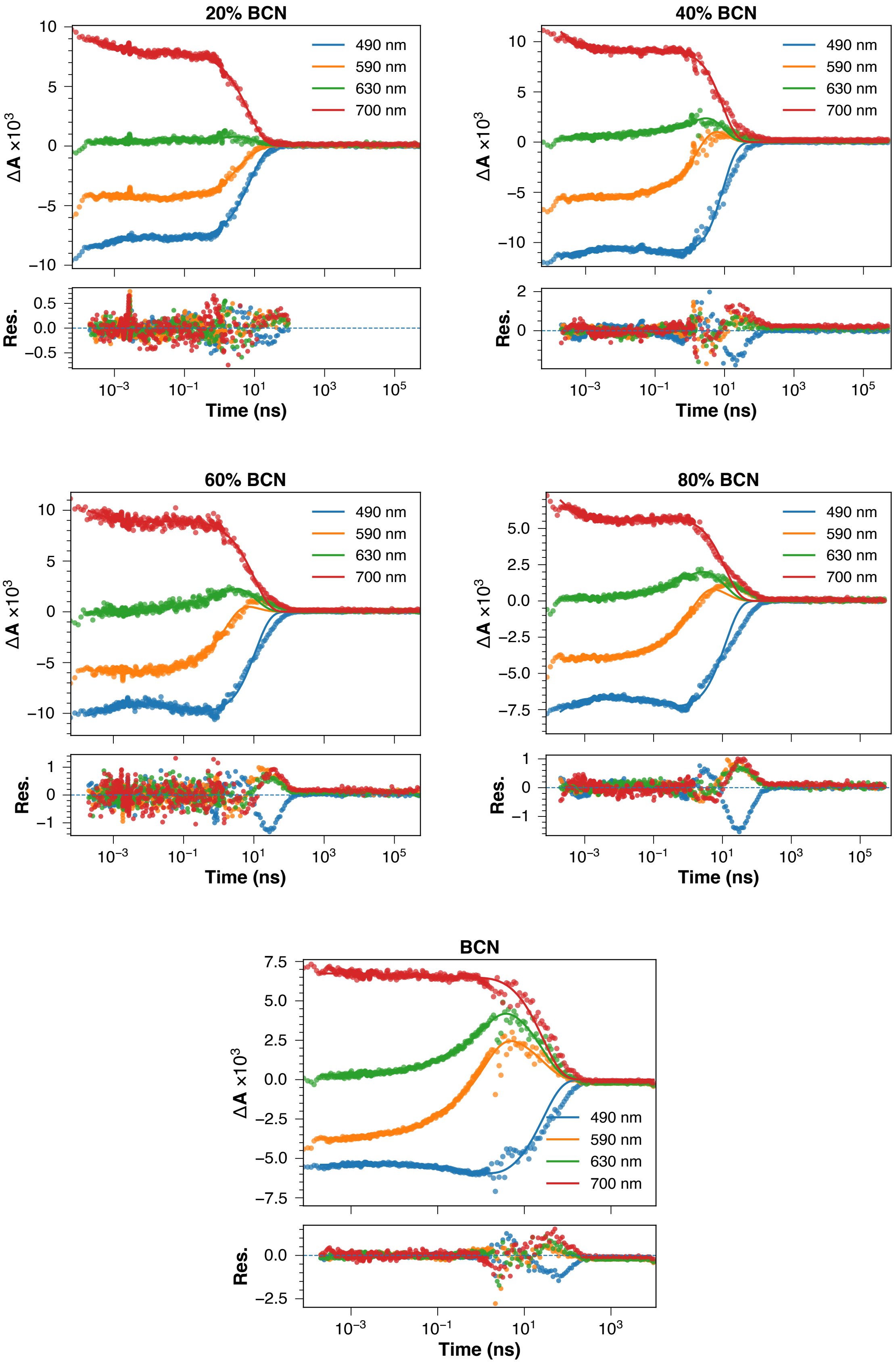

Figure S11: Upper panel: Transient absorption at selected wavelengths (circles) measured with **Cage** in TOL/BCN mixtures and best fits (solid lines). Lower panel: Residuals.

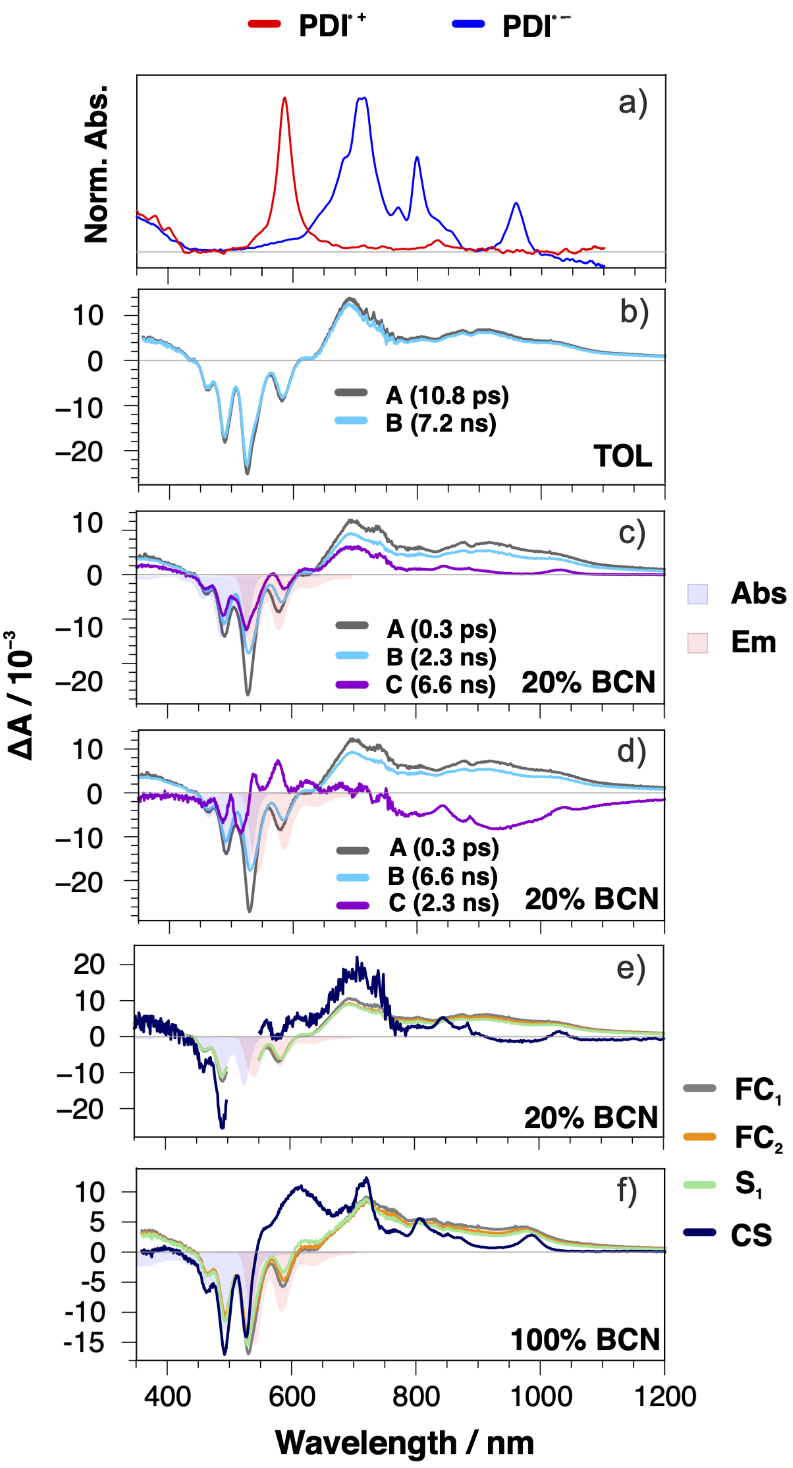

Figure S12: a) Stationary absorption spectra of $PDI^{\bullet+}$and $PDI^{\bullet-}$ taken from ref. 1,2. b) Evolution-associated difference spectra and time constants obtained from global analysis of the merged transient absorption data measured with **Cage** in TOL assuming a series of two successive exponential steps (A $\rightarrow$ B$\rightarrow$). c,d) same as b) but in 80:20 TOL/BCN mixture assuming three successive exponential steps (A $\rightarrow$ B$\rightarrow$ C$\rightarrow$), with increasing time constants (c) or with inverted kinetics (d). e,f) Species-associated difference absorption spectra obtained from a constraint target analysis of the data measured with **Cage** in 80:20 TOL/BCN mixture (e) and pure BCN (f).

S3.4 Quantum chemical calculations

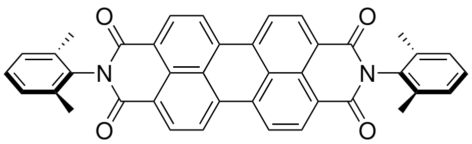

Figure S13: Structure of the PDI analogue used for calculations.

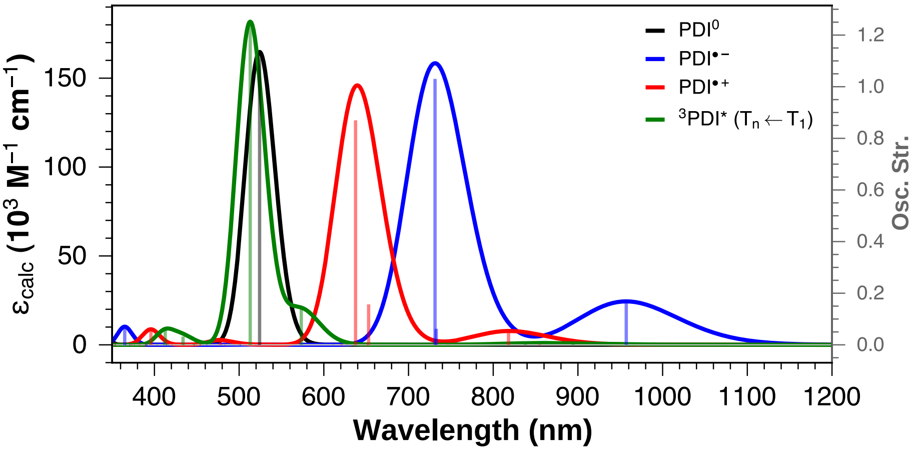

Figure S14: TD-DFT calculated electronic absorption spectra of the relevant species derived from the PDI model compound (Figure **S13**). The solid lines represent the convolved spectra (*left scale*), whilst the vertical bars indicate the oscillator strengths (*right scale*).

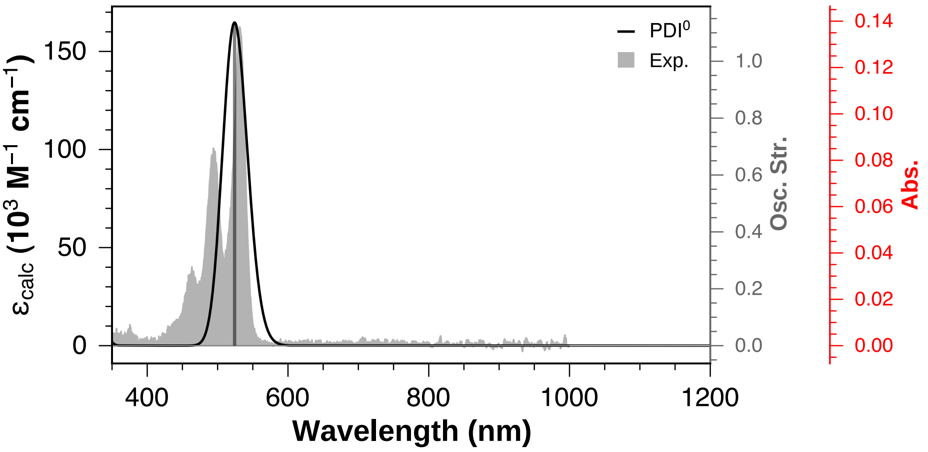

Figure S15: Comparison of the experimental and TD-DFT calculated electronic absoprtion spectra of the PDI model compound (Figure **S13**). The solid lines represent the convolved spectra (*left scale*), the vertical bars indicate the oscillator strengths.

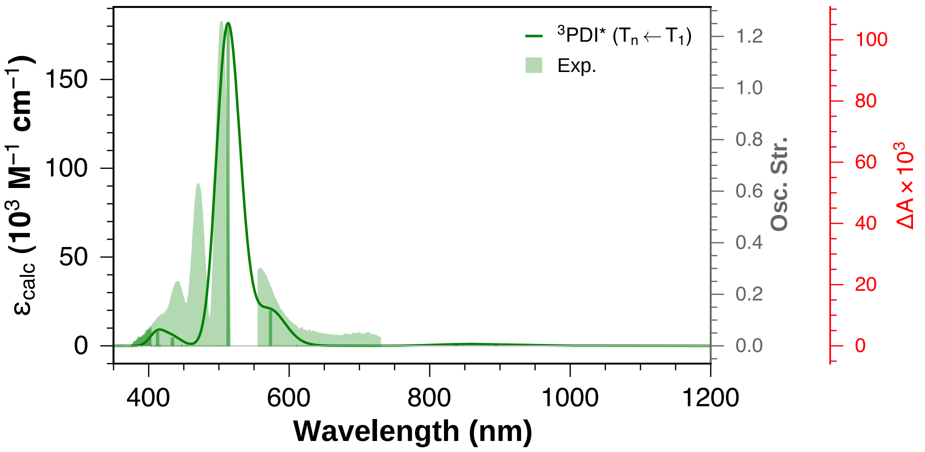

Figure S16: Comparison of the experimental absorption spectrum of the triplet excited state of the commercial PDI compound (Figure **S4**) in ACN measured by transient-absorption spectroscopy,[3] and of the TD-DFT calculated spectrum of the ^{3}PDI* model compound (Figure **S13**). The solid lines represent the convolved spectra (*left scale*), the vertical bars indicate the oscillator strengths.

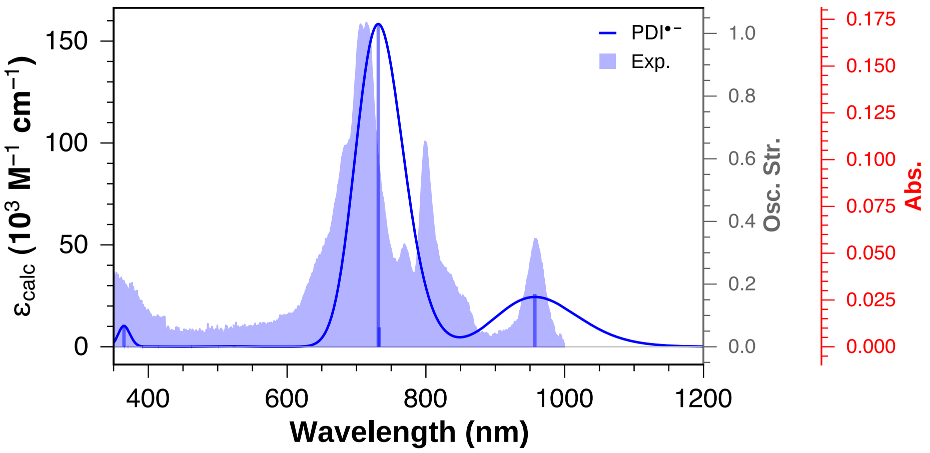

Figure S17: Comparison of the experimental absorption spectrum of the commercial PDI$^{\bullet-}$ (Figure **S4**) in BCN measured using spectroelectrochemistry and of the TD-DFT calculated spectra of the PDI$^{\bullet-}$ model compound (Figure **S13**). The solid lines represent the convolved spectra (*left scale*), the vertical bars indicate the oscillator strengths.

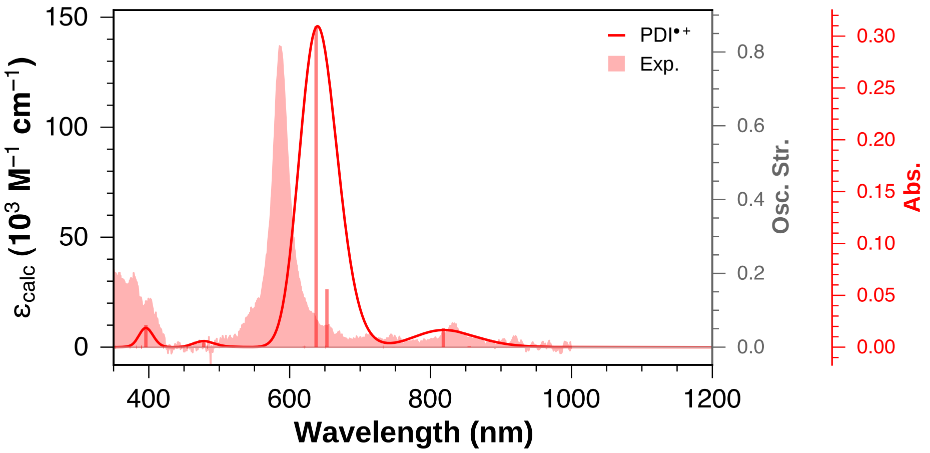

Figure S18: TD-DFT calculated spectra of PDI$^{\bullet+}$ from the PDI model compound (Figure **S13**). The solid lines represent the convolved spectra (*left scale*), the vertical bars indicate the oscillator strengths, and the experimental spectrum of the commercial PDI compound (Figure **S4**) in ACN measured using spectroelectrochemistry is shown for comparison (*right scales*).

S3.5 Molecular dynamics simulations

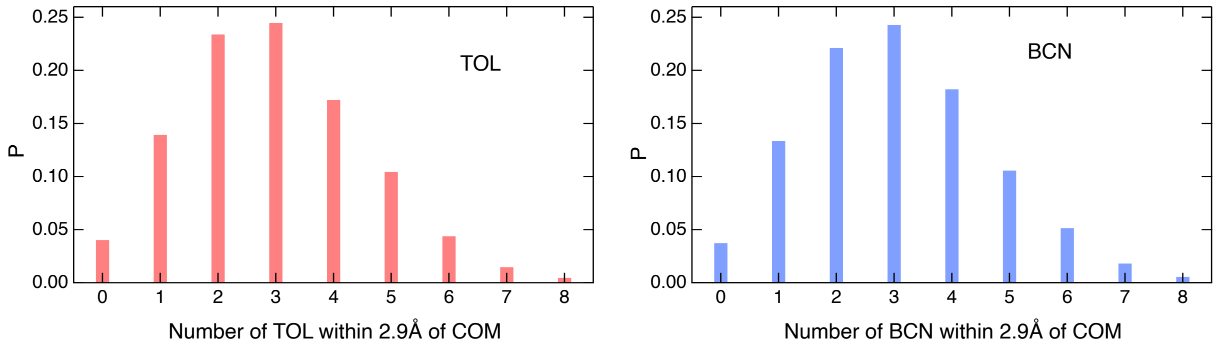

Figure S19: Histograms of the number of solvent molecules within 2.9 Å of the centre of mass (COM) of **Cage** from MD simulations in toluene (left) and benzonitrile (right). **Cage** can accommodate up to four TOL or BCN molecules entirely. Larger values in the histograms correspond to cases where some solvent molecules are not entirely located inside **Cage**.

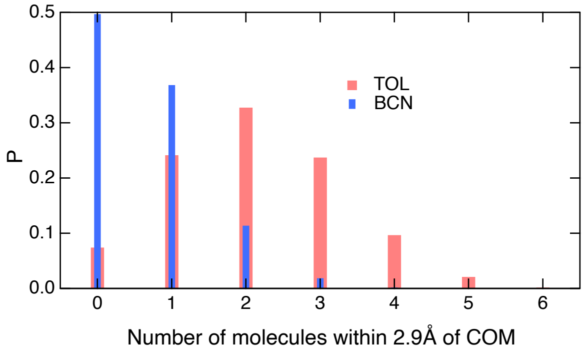

Figure S20: Histogram of the number of benzonitrile (BCN) and toluene (TOL) molecules within 2.9 Å of the centre of mass of **Cage** from MD simulations in a 80:20 (v/v) TOL/BCN mixture.

S4. References

[1] J. Kong, W. Zhang, G. Li, D. Huo, Y. Guo, X. Niu, Y. Wan, B. Tang, and A. Xia, Excited-State Symmetry-Breaking Charge Separation Dynamics in Multibranched Perylene Diimide Molecules, J. Phys. Chem. Lett. **11**, 10329 (2020).

[2] H.-H. Huang, K. S. Song, A. Prescimone, A. Aster, G. Cohen, R. Mannancherry, E. Vauthey, A. Coskun, and T. Šolomek, Porous shape-persistent rylene imine cages with tunable optoelectronic properties and delayed fluorescence, Chem. Sci. **12**, 5275 (2021).

[3] D. H. Cruz Neto, E. Sucre-Rosales, and E. Vauthey, Making Charge Recombination Spin-Forbidden for Efficient Generation and Excitation of Perylene Diimide Radical Anion by Pump–Pump–Probe Spectroscopy, J. Phys. Chem. Lett. **16**, 13241 (2025).

[4] A. Aster, F. Zinna, C. Rumble, J. Lacour, and E. Vauthey, Singlet Fission in a Flexible Bichromophore with Structural and Dynamic Control, J. Am. Chem. Soc. **143**, 2361 (2021).

[5] A. Aster, G. Licari, F. Zinna, E. Brun, T. Kumpulainen, E. Tajkhorshid, J. Lacour, and E. Vauthey, Tuning symmetry breaking charge separation in perylene bichromophores by conformational control, Chem. Sci. **10**, 10629 (2019).

[6] B. Lang, S. Mosquera-Vázquez, D. Lovy, P. Sherin, V. Markovic, and E. Vauthey, Broadband ultraviolet-visible transient absorption spectroscopy in the nanosecond to microsecond time domain with sub-nanosecond time resolution, Rev. Sci. Instrum. **84**, 73107 (2013).

[7] M. J. Frisch, G. W. Trucks, H. B. Schlegel, G. E. Scuseria, M. A. Robb, J. R. Cheeseman, G. Scalmani, V. Barone, G. A. Petersson, H. Nakatsuji, X. Li, M. Caricato, A. V. Marenich, J. Bloino, B. G. Janesko, R. Gomperts, B. Mennucci, H. P. Hratchian, J. V. Ortiz, A. F. Izmaylov, J. L. Sonnenberg, D. Williams-Young, F. Ding, F. Lipparini, F. Egidi, J. Goings, B. Peng, A. Petrone, T. Henderson, D. Ranasinghe, V. G. Zakrzewski, J. Gao, N. Rega, G. Zheng, W. Liang, M. Hada, M. Ehara, K. Toyota, R. Fukuda, J. Hasegawa, M. Ishida, T. Nakajima, Y. Honda, O. Kitao, H. Nakai, T. Vreven, K. Throssell, J. A. Montgomery Jr., J. E. Peralta, F. Ogliaro, M. J. Bearpark, J. J. Heyd, E. N. Brothers, K. N. Kudin, V. N. Staroverov, T. A. Keith, R. Kobayashi, J. Normand, K. Raghavachari, A. P. Rendell, J. C. Burant, S. S. Iyengar, J. Tomasi, M. Cossi, J. M. Millam, M. Klene, C. Adamo, R. Cammi, J. W. Ochterski, R. L. Martin, K. Morokuma, O. Farkas, J. B. Foresman, and D. J. Fox, Gaussian 16 rev. b.01, (2016).

[8] T. Yanai, D. P. Tew, and N. C. Handy, A new hybrid exchange-correlation functional using the coulomb-attenuating method (cam-b3lyp), Chem. Phys. Lett. **393**, 51 (2004).

[9] A. Hellweg and D. Rappoport, Development of new auxiliary basis functions of the karlsruhe segmented contracted basis sets including diffuse basis functions (def2-svpd, def2-tzvppd, and def2-qvppd) for ri-mp2 and ri-cc calculations, Phys. Chem. Chem. Phys. **17**, 1010 (2015).
[10] S. Grimme, S. Ehrlich, and L. Goerigk, Effect of the damping function in dispersion corrected density functional theory, J. Comput. Chem. **32**, 1456 (2011).
[11] M. J. Abraham, T. Murtola, R. Schulz, S. Páll, J. C. Smith, B. Hess, and E. Lindahl, Gromacs: High performance molecular simulations through multi-level parallelism from laptops to supercomputers, SoftwareX **1-2**, 19 (2015).
[12] A. W. Sousa da Silva and W. F. Vranken, Acpype - antechamber python parser interface, BMC Res. Notes **5**, 367 (2012).
[13] J. Wang, R. M. Wolf, J. W. Caldwell, P. A. Kollman, and D. A. Case, Development and testing of a general amber force field, J. Comput. Chem. **25**, 1157 (2004).
[14] L. E. Chirlian and M. M. Francl, Atomic charges derived from electrostatic potentials: A detailed study, J. Comput. Chem. **8**, 894 (1987).
[15] D. van der Spoel, M. M. Ghahremanpour, and J. A. Lemkul, Small molecule thermochemistry: A tool for empirical force field development, J. Phys. Chem. A **122**, 8982 (2018).
[16] T. Darden, D. York, and L. Pedersen, Particle mesh ewald: An n x log(n) method for ewald sums in large systems, J. Chem. Phys. **98**, 10089 (1993).
[17] B. Hess, H. Bekker, H. J. C. Berendsen, and J. G. E. M. Fraaije, Lincs: A linear constraint solver for molecular simulations, J. Comput. Chem. **18**, 1463 (1997).
[18] W. G. Hoover, Canonical dynamics: Equilibrium phase-space distributions, Phys. Rev. A **31**, 1695 (1985).
[19] M. Bernetti and G. Bussi, Pressure control using stochastic cell rescaling, J. Chem. Phys. **153**, 114107 (2020).
[20] R. J. Fernández-Terán, E. Sucre-Rosales, L. Echevarria, and F. E. Hernández, A Sweet Introduction to the Mathematical Analysis of Time-Resolved Spectra and Complex Kinetic Mechanisms: The Chameleon Reaction Revisited, J. Chem. Educ. **99**, 2327 (2022).
[21] M. N. Berberan-Santos and J. M. G. Martinho, The integration of kinetic rate equations by matrix methods, J. Chem. Educ. **67**, 375 (1990).
[22] J. S. Beckwith, C. A. Rumble, and E. Vauthey, Data analysis in transient electronic spectroscopy – an experimentalist's view, Int. Rev. Phys. Chem. **39**, 135 (2020).
